# Extending differential gene expression testing to handle genome aneuploidy in cancer

**DOI:** 10.1371/journal.pcbi.1014134

**Published:** 2026-03-27

**Authors:** Katsiaryna Davydzenka, Giulio Caravagna, Guido Sanguinetti

**Affiliations:** 1 Theoretical and Scientific Data Science Group, International School of Advanced Studies, Trieste, Italy; 2 University Campus Bio-Medico of Rome, Rome, Italy; 3 Department of Mathematics, Informatics and Geosciences, University of Trieste, Trieste, Italy; 4 Area Science Park, Trieste, Italy; Johns Hopkins University, UNITED STATES OF AMERICA

## Abstract

Genome aneuploidy, characterized by copy number variations (CNVs), profoundly alters gene expression in cancer through direct gene dosage effects and indirect compensatory regulatory mechanisms. However, existing differential gene expression (DGE) testing methods do not differentiate between these mechanisms, conflating all expression changes, limiting biological interpretability and obscuring key genes involved in tumor progression. To address this, we developed DeConveil, a computational framework that extends traditional DGE analysis by integrating CNV data. Using a generalized linear model with a negative binomial distribution, DeConveil models RNA-seq expression counts while accounting for copy number gene dosage effects. We proposed a more fine-grained gene decomposition into dosage-sensitive (DSGs), dosage-insensitive (DIGs), and dosage-compensated (DCGs), which explicitly de-couples changes due to CNVs and bona fide changes in transcriptional regulation. Analysis of TCGA datasets from aneuploid solid cancers resulted in notable reclassification of genes, refining and expanding upon the results from conventional methods. Functional enrichment analysis identified distinct biological roles for DSGs, DIGs, and DCGs in tumor progression, immune regulation, and cell adhesion. In a breast cancer case study, DeConveil’s CN-aware analysis facilitated the identification of both known and novel prognostic biomarkers, including lncRNAs, linking gene expression signatures to survival outcomes. Utilizing these biomarkers for each gene group significantly improved patient risk stratification, yielding more accurate predictions compared to conventional methods. These results highlight DeConveil’s ability to disentangle CNV-driven from regulatory transcriptional changes, enhancing gene classification and biomarker discovery. By improving transcriptomic analysis, DeConveil provides a powerful tool for cancer research, precision oncology, with potential applications in therapeutic target identification.

## Author summary

Identifying genes whose expression changes in cancer is fundamental to understand disease aetiology and to propose therapeutic targets. However, alterations to the copy number of genes, due to amplification or deletion events, can represent a significant confounder to differential expression quantification. Here we propose a simple model to correct for this confounder, identifying a finer characterization of coordinated changes in gene expression and copy number. We show on a number of data sets that this new characterization has prognostic value and sheds light on gene regulation in cancer.

## Introduction

Cancer is a highly heterogeneous disease characterized by extensive genomic alterations with DNA CNVs and genome aneuploidy emerging as defining hallmarks across most tumor types [1–3]. CNVs represent structural changes, such as gains or losses of specific chromosomal segments, which profoundly reshape the transcriptional landscape of cancer cells [4–6]. These changes can create gene dosage effects, amplifying or reducing mRNA transcript levels for genes within the affected regions [7,8]. Such disruptions have profound consequences for tumor progression, driving tumorigenesis [9], facilitating metastasis [10], and contributing to therapy resistance [11].

However, the relationship between somatic CNVs and gene expression is complex [4,12–19]. While some genes in altered regions exhibit expression changes that correlate with CNVs, such as oncogenes in amplified regions or tumor suppressors in deleted regions [12], many others exhibit only moderate or no expression changes [12–15], suggesting the involvement of additional regulatory mechanisms. For example, Zhou et al. (2017) [13] identified CNV-driven differentially expressed genes (DEGs) in hepatocellular carcinoma, highlighting CNVs as key drivers of transcriptional dysregulation. Mohanty et al. (2021) [19] further emphasize that CNVs alone do not dictate expression changes in aneuploid cancers; instead, gene-specific regulatory dynamics and compensatory mechanisms can modulate these effects. This complexity underscores the need for advanced statistical approaches to distinguish CNV-driven expression changes from independent regulatory alterations.

DGE analysis remains fundamental for studying transcriptomic alterations in cancer, identifying key oncogenic pathways, therapeutic targets, and biomarkers [20,21]. Widely used statistical tools for DGE analysis, such as DESeq2 [22], edgeR [23], and limma [24] employ statistical models that effectively handle RNA-seq count data, assuming gene expression changes arise solely from biological or experimental factors. However, these methods do not account for CNVs, implicitly assuming that the genes have the same CNVs across samples (or no CNVs) overall. This assumption is problematic in cancer, where aneuploidy introduces widespread CNVs that can drive gene expression changes. A key limitation is the inability to determine whether observed expression changes result from CNVs or other regulatory mechanisms. This can create significant challenges for interpreting DGE results in cancer studies, potentially obscuring key biological insights and misleading conclusions. While some progress has been made in integrating CNV data into other genomic contexts, such as DNA methylation, similar advancements in transcriptomics remain limited. For example, the ABCD-DNA tool integrates CNV data to enhance the analysis of DNA methylation [25]. However, a parallel framework for transcriptomics is still lacking.

To address these limitations, we developed DeConveil, a computational framework that explicitly integrates CNV effects into DGE analysis. DeConveil extends traditional statistical models by incorporating CNV data using a generalized linear model (GLM) with a negative binomial (NB) distribution, allowing for a more detailed interpretation of gene expression changes. This approach refines gene classification by disentangling genes whose expression is primarily driven by CNVs from those regulated through other biological mechanisms.

Application of DeConveil to aneuploid cancer datasets demonstrates its broad utility and capacity to uncover shared and specific mechanisms across cancers. In a case study on breast cancer, DeConveil provided a more refined categorization of genes based on their relationship to gene CNVs, including novel long non-coding RNAs (lncRNAs). This refined classification not only facilitated the identification of potential prognostic genes but also provided a deeper understanding of their biological roles and regulatory mechanisms underlying each gene category.

## Results

### DeConveil approach

**Fig 1:**
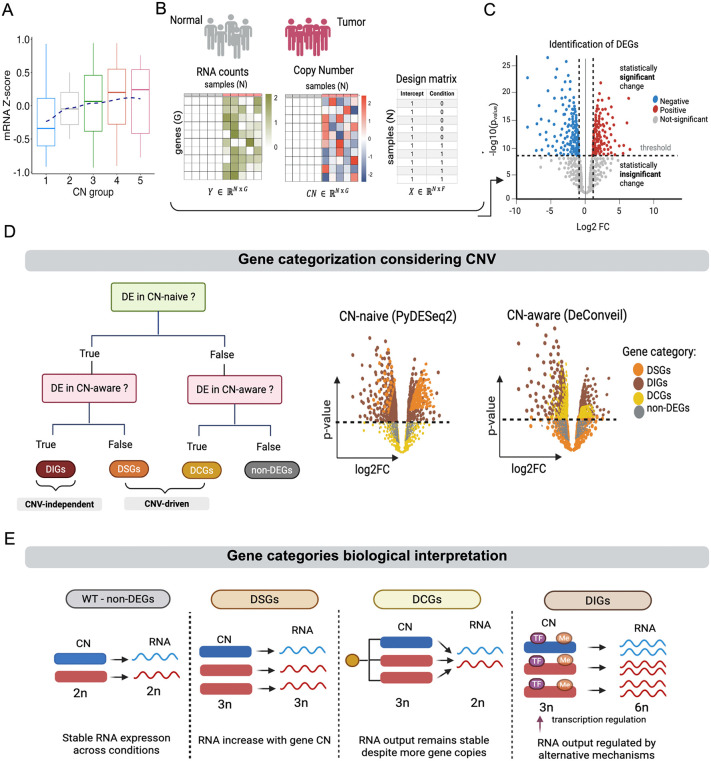
Overview of the DeConveil framework. **(A)** Relationship between gene expression and DNA CN. Boxplots show the distribution of mRNA Z-scores across five CN groups in LUAD tumor samples; the dashed blue line represents a locally weighted scatterplot smoothing (LOESS) fit. (**B)** Input data and modeling design. Matched RNA-seq read counts and absolute gene CN values are provided as input matrices; a design matrix encodes sample conditions (e.g., tumor = 1, normal = 0). **(C)** Differential expression testing. Volcano plot illustrates selection of DEGs based on |log_2_FC| > 1 and p-value < 0.05. (**D)** Gene classification framework. Comparison of CN-naive (PyDESeq2) and CN-aware (DeConveil) models assigns genes to dosage-sensitive (DSGs), dosage-insensitive (DIGs), dosage-compensated (DCGs), or non-DEGs categories. **(E)** Conceptual summary of gene-dosage classes. DSGs show CN-dependent expression, DIGs show CN-independent regulation, DCGs exhibit buffered responses to CN alterations, and non-DEGs show stable expression. Fig 1E was created in BioRender. Davydzenka, **K.** (2026) https://BioRender.com/9l2b19o.

The primary goal of DeConveil is to account for the influence of gene CNVs on RNA transcripts counts, when analyzing gene expression differences between contrasted conditions or sample groups. Our strategy is based on a GLM, commonly adopted in RNA-seq differential expression (DE) testing [22,23]. DeConveil models transcript abundance using NB regression that explicitly incorporates gene- and sample-specific CN dosage as a scaling factor (see Methods). This enables correction for CN-driven expression shifts, which are particularly relevant in cancer, as observed in different aneuploid cancer types where transcript levels are proportional to CN states (Figs 1A, S1 (A)).

The DeConveil framework integrates three key data layers (Fig 1B): RNA-seq read counts, absolute CN profiles, and a sample specific design matrix encoding experimental conditions. These inputs are used in our statistical framework to perform the log₂ fold change (log₂FC) and p-values calculations in order to perform gene classification. Genes are first classified as DE if they meet both statistical significance and effect size thresholds: adjusted p-value < 0.05 and |log₂FC| > 1 (Fig 1C). Genes that do not meet these criteria are labeled as non-differentially expressed (non-DEGs).

To disentangle regulatory-driven expression changes from those driven by CN dosage, DeConveil compares outputs from a standard CN-naive model (PyDESeq2 [26]) and its CN-aware (DeConveil) counterpart. Based on this comparison, genes are classified into four biologically interpretable categories (Fig 1D, 1E):

1. Dosage-sensitive genes (DSGs): these show RNA expression levels that scale proportionally with gene CN, consistent with gene dosage principles. While this linear assumption simplifies modeling, it may not capture all transcriptional complexities.
2. Dosage-insensitive genes (DIGs): these exhibit DE that cannot be explained by CN changes alone. Instead, their expression shifts are likely driven by regulatory mechanisms such as transcription factor activity, epigenetic alterations, or post-transcriptional control [19].
3. Dosage-compensated genes (DCGs): in these genes the changes in gene CN do not linearly affect gene expression. This occurs because cells employ regulatory mechanisms to buffer these changes to maintain transcriptional homeostasis. Recent studies in cancer have demonstrated the presence of dosage compensation [14,26–30]. For instance, when CN increases, cells may reduce transcription from amplified genes to prevent overexpression. Conversely, when CN decreases, cells may upregulate transcription to compensate for gene loss.
4. Non-DEGs: genes showing no statistically significant RNA level changes, likely reflecting transcriptional stability under the tested conditions.

This classification scheme enables more refined biological interpretation of expression changes.

The computational framework is implemented in Python as an extension of the DESeq2/PyDESeq2 statistical pipeline (https://github.com/caravagnalab/DeConveil).

### DeConveil validation using simulated data

**Fig 2:**
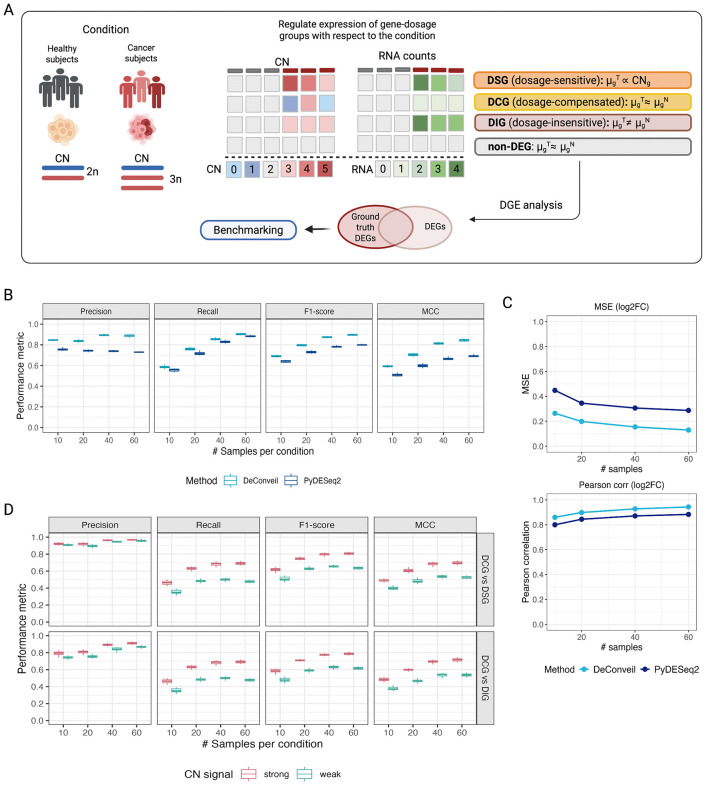
DeConveil benchmarking on simulated gene expression data. **(A)** Schematic overview of the simulation framework. Gene expression counts are generated for two biological conditions (e.g., healthy vs tumor) with CN alterations present in one condition. Expression differences in each gene-dosage class (DSGs, DCGs, DIGs, non-DEGs) reflect changes in the expected mean expression µ_g_, as defined by the generative model (see S1 Text), which jointly depends on biological condition and CN. Ground-truth DEGs are defined by the simulation and compared against detected DEGs. (**B)** Evaluation of DE detection performance under CN confounding. Precision, recall, F1-score, and Matthews correlation coefficient (MCC) are shown as a function of sample size per condition (10, 20, 40, and 60), comparing DeConveil (CN-aware) and PyDESeq2 (CN-naive). **(C)** Assessment of DeConveil’s accuracy in effect size estimation. Top: Mean Square Error (MSE) between estimated and true log₂FC. Bottom: Pearson correlation between estimated and true log₂FC. Results compare DeConveil and PyDESeq2. (**D)** Gene dosage classification performance of DeConveil. Precision, recall, F1-score, and MCC are shown for distinguishing DCGs from DSGs and DIGs, under weak and strong CN signal conditions, as a function of sample size.

We first asked whether CN-aware modeling can correct CN-driven confounding in DE analysis. We evaluated DeConveil using simulated RNA-seq counts data with known ground truth, generated under controlled CN perturbations across varying sample sizes (see Methods: Simulation benchmarking). Genes were assigned to distinct dosage classes reflecting different relationships between CN and expression (Fig 2A), including DSGs, DIGs, DCGs and non-DEGs. In this simulation framework, DIGs represent true condition-dependent DE independent of CN, whereas DSGs exhibit expression changes driven by CNV and thus constitute structured false positives in CN-naive DE analysis. Non-DEGs genes are unaffected by both condition and CN. DCGs represent a distinct and more challenging class: these genes are truly DE but appear as false negatives in CN-naive model due to compensatory regulation. We therefore evaluated their recovery and classification in a separate analysis.

We compared DeConveil to CN-naive DE method PyDESeq2. DE detection performance was evaluated using precision, recall, F1-score, and Matthews correlation coefficient (MCC) [31], treating DIGs as true positives and DSGs and non-DEGs as true negatives. Across all sample sizes, DeConveil consistently outperformed the CN-naive approach, demonstrating separation of truly DE genes from CN-driven false positives (Fig 2B). Specifically, DeConveil achieved higher scores in precision (0.84–0.89 vs. 0.78–0.74), recall (0.58–0.90 vs. 0.55–0.88), F1-score (0.68-0.90 vs. 0.64–0.80), and MCC (0.59-0.85 vs. 0.52–0.70). (Fig 2B). These performance gains became more pronounced in larger sample sizes.

We further assessed the impact of CN-awareness on effect size estimation. Estimated log₂FC were compared with the known ground truth values used during simulation (Fig 2C). DeConveil achieved lower mean squared error (MSE; 0.26-0.13 vs 0.45-0.29) and higher Pearson correlation (R² = 0.86-0.94 vs 0.80-0.88) across all sample sizes, indicating more accurate recovery of DE effect sizes. These results demonstrate that DeConveil effectively corrects CN-induced confounding in DE analysis.

Beyond DE detection, we evaluated whether DeConveil can distinguish distinct gene dosage behaviors when CN effects are explicitly modeled. Using the simulated ground truth, we assessed DeConveil’s ability to classify DCGs against DSGs and DIGs under varying CN signal strengths. Classification performance was quantified using precision, recall, F1-score, and MCC. Although recall and MCC were comparable between the DCGs vs DSGs and DCGs vs DIGs classification tasks, systematic differences were observed in precision (Fig 2D). Precision was consistently higher and more stable when distinguishing DCGs from DSGs (0.91-0.96) than from DIGs (0.76-0.89), indicating more frequent false positive DCGs assignments in the presence of DIGs. This may reflect partial overlap between condition-dependent expression changes and dosage compensation effects. As expected, overall classification performance improved with increasing sample size and stronger CN signal, while performance under weak CN signal was reduced, reflecting intrinsic limitations in signal identifiability.

Additional analysis of the confusion matrices and performance metrics in S2 Fig (C, E) showed higher and more stable accuracy for DIGs and DSGs than for DCGs. Across increasing sample sizes, DIGs and DSGs classes achieved precision of 0.79-0.85 and 0.82-0.95 with recall of 0.53-0.88 and 0.31-0.70, respectively, whereas DCGs performance was lower (precision 0.62-0.80; recall 0.40-0.58). Errors were dominated by false negatives, and performance improved with sample size, consistent with limited signal rather than model misspecification.

To test DeConveil’s robustness against CN input uncertainty, we introduced increasing levels of noise (10–25%) to the CN matrix entries and tested performance across different sample sizes (10–60). We used three metrics to assess stability (S3 Fig): mean Jaccard index to measure consistency in gene group assignments, Pearson correlation (R²) for log₂FC estimates, and Spearman correlation (R²) for adjusted p-value rankings. Overall, DeConveil demonstrated strong robustness to CN noise, particularly for DIGs and non-DEGs groups. These groups maintained stable classification and accurate effect size estimates across all conditions (Jaccard index > 0.85, R² > 0.75), even at higher noise levels and larger sample sizes. In contrast, CN-sensitive groups (DSGs and DCGs), which are more dependent on accurate CN information, exhibited moderate declines in performance metrics under noise. This is particularly evident in Jaccard index and Spearman correlation MCC metric.

Additional analyses in S2 Fig (A, B) confirms proper statistical calibration and supports the choice of multiple-testing correction (S2 Fig (C-F)) strategy used throughout this study. Consistent with these results, application of the same FDR test framework to multiple TCGA cancer cohorts reduced ambiguous gene assignments relative to independent BH corrections (S4 Fig).

Overall, these results demonstrate that incorporating CN information, enables DeConveil to mitigate the CN-driven confounding in bulk RNA-seq analysis while providing robust and interpretable gene dosage classification.

### Application of DeConveil to DGE analysis

We used DeConveil to understand how CN corrections of gene expression influence DGE analysis results outcomes in a real scenario. We focused on evaluating the DeConveil ability to categorize gene expression based on our hypothesis of transcriptional effects driven by CNVs. For this analysis, we selected different solid cancer types with high variability in gene CN based on availability of matched normal-tumor samples.

Using a set of 45 tumor-normal matched samples of lung adenocarcinoma (LUAD), we compared PyDESeq2 and DeConveil approaches to demonstrate how our CN-informed approach enables more refined gene expression categorization by distinguishing active expression changes from passive effects caused by CNVs. To enhance the reliability of the results, we excluded genes with low expression in normal tissue (mean expression < 10 reads). After filtering, our analysis focused on 19,830 genes.

**Fig 3:**
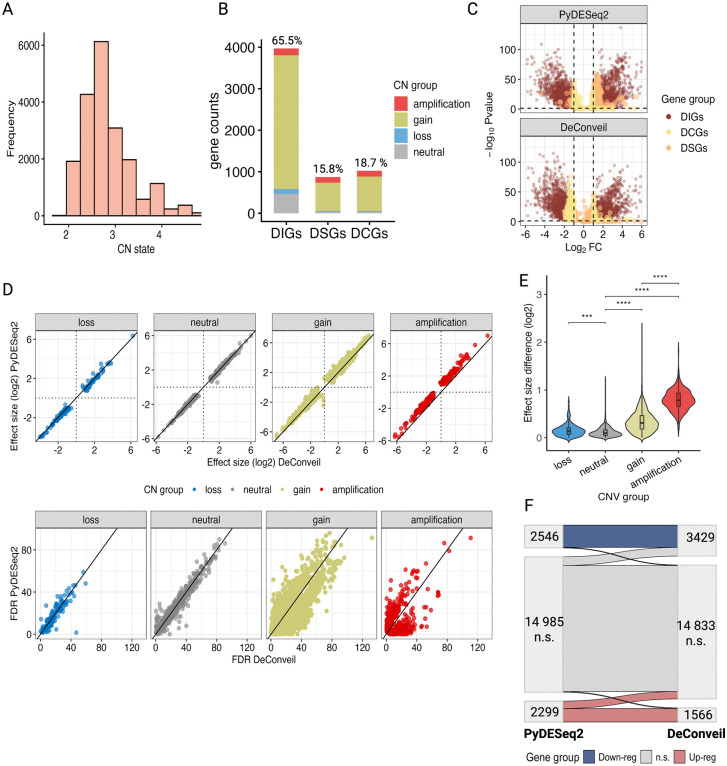
Impact of CN corrections on DGE analysis in lung adenocarcinoma (LUAD). **(A)** Distribution of CN states across LUAD tumor samples. (**B)** Gene categorization by CN status and DeConveil class (DSGs, DIGs, DCGs). Genes with CN loss (CN = 0 or 1 in ≥25% of samples) are explicitly shown here despite having near-diploid mean CN values. Stacked bars indicate proportions of CN states (loss, neutral, gain, amplification), with percentages denoting fractions of the total gene set. **(C)** Volcano plots comparing PyDESeq2 (CN-naive) and DeConveil (CN-aware) DE analyses. Genes are plotted by log₂FC and FDR; significance thresholds are |log₂FC| > 1 and FDR < 0.05**. (D)** Comparison of effect size (log₂FC) and FDR (bottom row) estimates between PyDESeq2 and DeConveil across CN states (loss, neutral, gain, and amplification). The diagonal reference line represents a one-to-one correlation. **(E)** Distribution of effect size differences (log₂FC) between methods across CN states. **(F)** Sankey diagram showing reassignment of genes between expression categories (upregulated, downregulated, non-significant) when CN correction is applied.

Our results reveal that CN gains and amplifications (CN 3–5) have a significant impact on gene expression, affecting approximately 75% of DEGs (Fig 3A-3C). DeConveil identified 817 (15.8%) DSGs, 3391 (65.5%) DIGs, and 969 (18.7%) DCGs (Fig 3B). We then assessed the differences between PyDESeq2 and DeConveil analyses by comparing the effect sizes (log₂FC*)* and false discovery rate (FDR) across both methods (Fig 3D). For genes with neutral CNs (no CN change) used as controls, we observed concordance between the two methods (diagonal trend). However, genes affected by CN gains and amplifications exhibited higher deviations from the diagonal, confirming the influence of CN adjustment in regions with aneuploidy. Notably, DeConveil analysis increased FDR for amplified and gain-affected genes (Fig 3D). On average, FDRs shifted by 0.0033, with 40.75% (1837 genes) showing increased p-values, reinforcing this trend. Furthermore, 122 genes lost statistical significance (p < 0.05 in CN-naive but p > 0.05 in CN-aware analysis).

To evaluate expression variability, we analyzed effect size differences between the methods (Fig 3E). Amplified and gain genes showed the largest effect size recalibrations of 0.79 ± 0.28 and 0.34 ± 0.26 respectively, while neutral and loss categories exhibited minimal changes (0.11 ± 0.12 and 0.14 ± 0.14) indicating less CN-driven effect. For instance, the number of downregulated genes increases after CN-aware adjustment (from 2526 to 3431), while the number of upregulated genes decreases (from 2318 to 1567), highlighting DeConveil’s ability to separate active regulatory effects from passive CNV-driven changes (Fig 3F).

We extended this analysis to four other cancer types with varying levels of CN variability (S5 and S6 Figs): lung squamous cell carcinoma (LUSC), breast invasive carcinoma (BRCA), liver hepatocellular carcinoma (LIHC), and kidney renal clear cell carcinoma (KIRC). Cancers with higher CN variability (CN 1–5), such as LUSC, BRCA, and LIHC, showed a greater impact of CN corrections, as evidenced by a larger proportion of DSGs (14.4 - 24.4%) and DCGs (11.3 - 20.9%) (S5 Fig (A-C)). In contrast, KIRC, which exhibits lower CN variability (CN 1–3), underwent minimal gene classification shifts, with fewer DSGs (11.1%) and DCGs (9.4%). This suggests that the influence of CN corrections is less pronounced in cancers with low CN variability.

### Insights into gene-dosage classes in aneuploid cancers

**Fig 4:**
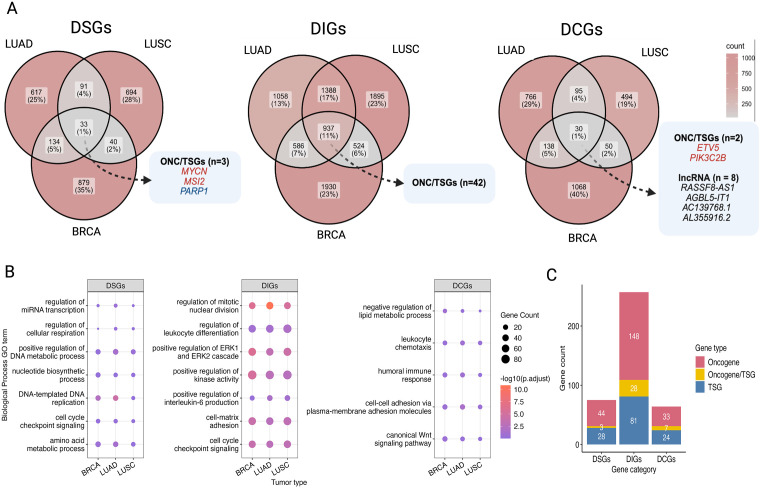
Cross-cancer comparison of gene-dosage classes and their functional associations. **(A)** Venn diagrams illustrate the overlap of DeConveil defined gene categories (DSGs, DIGs, and DCGs) across LUAD, LUSC, and BRCA. Selected oncogenes (ONC) and tumor suppressor genes (TSGs) found in each category are highlighted. Additionally, lncRNAs were identified within the DCGs category. Genes classified differently across cancer types are assigned to all relevant categories. **(B)** Gene Ontology (GO) over-representation analysis for biological processes associated with DSGs, DIGs, and DCGs across three cancer types. Dot size indicates the number of genes per term, and color denotes enrichment significance (–log10 adjusted p-value). **(C)** Distribution of ONC and TSGs within each gene category across private DEGs of three cancer types.

We further analyzed DSGs, DIGs, and DCGs identified by DeConveil across three epithelial origin aneuploid cancers, LUAD, LUSC, and BRCA (Fig 4A), to explore shared and private gene expression patterns and uncover functional pathways associated with each gene category.

Among the DSGs, only 33 (0.53%) were shared across all three cancers, including key oncogenes (*MYCN* [32], *MSI2* [33]), and tumor suppressors (*PARP1* [34]), reported to be critical for tumor progression and survival. Most DSGs are cancer specific (S1 Table) across all analyzed tumor types (11% of genes are private of the total 15%).

DIGs, more abundant than DSGs, showed broader conservation across three cancers (S2 Table), with 937 (15.2% of the total 68.6%) shared DIGs, including 42 known oncogenes and tumor suppressors. Their greater stability suggests that DIGs represent a default expression state, largely independent of CNVs, allowing tumors to maintain essential pathways despite genomic alterations.

DCGs also showed the least overlap across cancers, with only 30 (0.5% of the total 16%) shared genes, many of which are lncRNAs. This low overlap, along with the presence of lncRNAs, suggests that DCGs may function as regulatory elements influencing oncogenes and tumor suppressors activity through epigenetic or transcriptional mechanisms.

To assess the functional relevance of these shared gene categories, we performed Gene Ontology (GO) over-representation analysis (Fig 4B). The analysis revealed that shared DSGs are enriched in metabolic and cell cycle processes, confirming their importance in cancer cell survival under dosage variability. DIGs are linked to cell cycle, immune response, and oncogenic signaling pathways, supporting their role in tumor maintenance beyond CNV effects. Meanwhile, DCGs were primarily linked to immune regulation and cell adhesion, suggesting their potential involvement in tumor-immune interactions.

Additionally, we examined the functional significance of private cancer-specific genes within each category. For instance, functional analysis (S7 Fig) linked DIGs to immune regulation and cell proliferation in LUAD, mesenchymal differentiation in LUSC, and hormone metabolism in BRCA, emphasizing their role in sustaining tumor-specific traits. Functional enrichment of private DCGs (S7 Fig) highlighted their involvement in tumor-immune interactions and metabolic adaptation, including MHC complex and T-cell activation in LUAD, cytokine regulation in LUSC, and insulin secretion in BRCA.

To further refine our understanding of the significance of private genes, we mapped them to known cancer-specific oncogenes and TSGs (Fig 4C). As expected, the DIG category contained the highest number of oncogenes (n = 172) and tumor suppressors (n = 110), compared to DSGs (n = 55 oncogenes, n = 53 TSGs) and DCGs (n = 53 oncogenes, n = 31 TSGs). This supports the idea that DIGs encompass a broader range of critical cancer genes, many of which may operate independently of CNV effects, relying instead on regulatory mechanisms for tumor progression.

To further characterize the behavior of DeConveil derived gene classes, we compared these classifications with results from a complementary tumor-only NB regression modeling approach (see S2 Text). DSGs showed high concordance across methods with agreement increasing under strong-effect thresholds (73–94%) (S8 Fig (A)). As expected, DIGs were predominantly reassigned to directional NB classes, mapping mainly to DSGs (73–81%) and to a lesser extent to DCGs (5–21%). In contrast, DCGs displayed lower concordance, most evident at moderate effect sizes (33%) and were preferentially reassigned to DSG NB class (64–82%) (S8 Fig (A, B)). Entropy-based stratification further indicated that DCGs represent a heterogeneous group with distinct dosage–response behaviors (S8 Fig (C)). Together, these results suggest that DCGs represent a biologically challenging class whose apparent discordance across models may reflect underlying regulatory heterogeneity rather than methodological inconsistency.

We provided illustrative examples of class-specific dosage behaviors using representative genes from LUAD. Expression-CN relationships follow expected patterns for each class (S9 Fig), with strong positive associations for DSGs and attenuated or more complex patterns for DIGs and DCGs.

### Identification of prognostic biomarkers in breast cancer using DeConveil

In this case study, we investigated the prognostic potential of DSGs, DIGs, and DCGs identified by DeConveil in BRCA dataset, a solid highly aneuploid tumor. From 110 paired tumor-normal samples, DE analysis identified 1073 (17.5%) DSGs, 3788 (61.6%) DIGs, and 1284 (20.9%) DCGs among 22 076 analyzed genes (S5 Fig (B)). Notably, most of these genes were primarily influenced by CN gains and amplifications.

**Fig 5:**
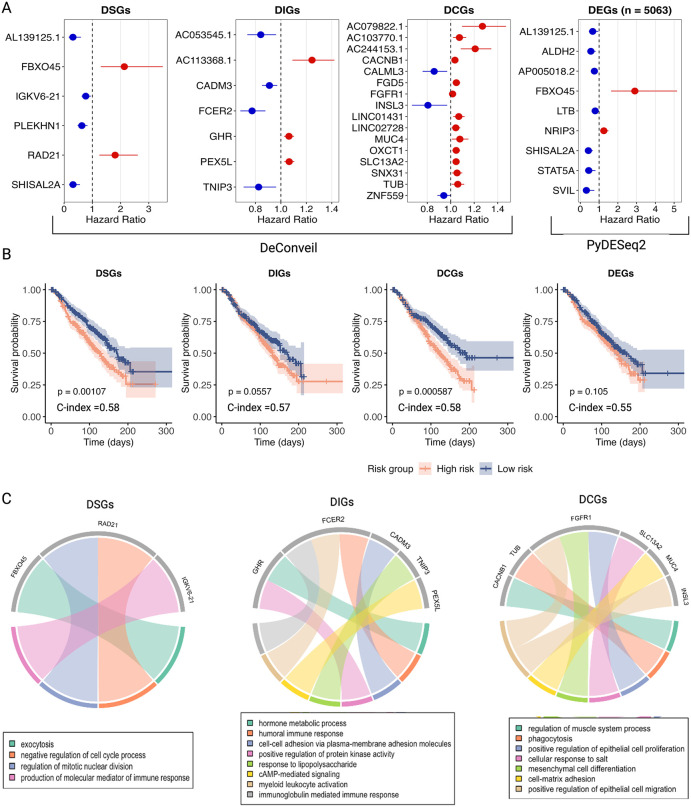
Prognostic relevance of DeConveil gene-dosage classes. **(A)** Cox proportional hazards analysis identified prognostic genes within DeConveil defined DSGs, DIGs, DCGs, compared with PyDESeq2 DEGs. Forest plots show hazard ratios (HR) for genes selected by LASSO regression (p < 0.05). (**B)** Kaplan-Meier survival curves comparing high-risk and low-risk patient groups based on prognostic gene signatures derived from DSGs, DIGs, and DCGs under DeConveil and PyDESeq2 models (p < 0.05). The concordance index (C-index) values indicate the predictive accuracy of the survival model. **(C)** GO enrichment analysis of biological pathways associated with DeConveil derived prognostic genes in each gene category.

To assess their prognostic relevance, we applied Cox proportional hazards model [35] to estimate hazard ratios (HRs) and confidence intervals (CIs). This analysis identified 142 DSGs, 161 DIGs, and 69 DCGs significantly associated with overall survival (p-value < 0.05). Further feature selection using LASSO regression highlighted key genes with prognostic potential. Several key genes emerged as significant biomarkers having prognostic potential (Fig 5A). For example, DSGs like *FBXO45* and *RAD21*, along with DIGs such as *AC113368.1*, *PEX5L*, and *GHR*, were associated with worse survival outcomes (HR > 1). Similarly, several DCGs, including *FGD5*, *FGFR1*, *MUC4*, *INSL3* displayed significant HRs. Many of these genes have well-established roles in breast cancer progression [36–39], metastasis [40, 41], and therapy resistance. Interestingly, the analysis also identified lncRNAs within the DCG category with high prognostic potential, such as *AC079822.1*, *AC244153.1*, and *LINC01431*. lncRNA is known as novel gene regulatory molecules involved in cancer metabolic reprogramming and progression [42].

A comparison between DeConveil and PyDESeq2 results (Fig 5A) further highlighted the advantages of DeConveil’s classification. The DeConveil identified a larger set of prognostic genes with moderate HRs, detecting key genes that PyDESeq2 overlooked. For example, among the genes with HR > 1, only one (*FBXO45*) was identified by both approaches. This demonstrates the increased sensitivity of the DeConveil CN-aware approach in identifying clinically relevant genes.

To assess the predictive utility of gene signatures from each category, we calculated prognostic scores and stratified an independent cohort of 520 breast cancer patients from the METABRIC dataset into high- and low-risk groups based on median scores. Kaplan-Meier survival analysis showed that DSGs and DCGs provided the most significant separation between risk groups, with p-values < 0.001 and < 0.0006, respectively (Fig 5B). DIGs also showed a clear separation between high- and low-risk groups, but the effect was less pronounced (p < 0.05). These results highlight the high prognostic value of DeConveil’s gene classification framework.

Pathway enrichment analysis of the identified gene signatures (Figs 5C, S10) further supported their functional distinctions. DSGs and DCGs, in particular, were enriched in key cancer hallmarks, including cell cycle regulation, proliferation, adhesion, migration, and mesenchymal cell differentiation. In contrast, the DIG signature was enriched in a broader range of cancer-specific processes, such as hormone metabolism, immune response, cell adhesion, and signaling. These functional distinctions help explain the prognostic significance of these gene signatures.

## Discussion

Cancer transcriptomic heterogeneity is shaped by the interplay between genomic alterations and transcriptional regulation. Traditional DGE analyses identify expression changes across conditions but fail to assess the effect of CNVs in explaining such variations. In the context of cancers, where CNVs are prevalent, this makes it hard to distinguish which regulatory processes are impacted by CNVs, potentially obscuring key biological mechanisms underlying tumor progression.

DeConveil extends canonical approaches for DGE with CNV data, allowing a better accounting of the role of CNVs in impacting gene expression. An immediate byproduct of this new approach is the possibility of classifying genes based on the interplay among transcriptional status and CNVs. This innovative classification reveals which genes are compensated by the CNVs and those sensitive or insensitive to aneuploidy. These hypothesised biological mechanisms suggest complex regulatory mechanisms that might back up changes in gene multiplicity, underlying a complicated post-transcriptional regulatory network that acts to disrupt tissue homeostasis in the context of cancer. The implications of this new approach are also statistical. Traditional CN-naive approaches often fail to report dosage-compensated genes, suggesting that our approach might improve even established analyses of large cohorts [42–46].

We used DeConveil to analyse TCGA datasets, identifying shared and cancer-type-specific patterns of DEGs. In breast cancer, we identified known and novel prognostic biomarkers, including previously uncharacterised lncRNAs. By stratifying these genes based on CNV-related expression mechanisms, the framework improved risk group stratification and outperformed traditional DGE methods in predicting survival outcomes. This refined prognostic approach could aid in personalised treatment planning by identifying high-risk patients who may benefit from targeted interventions.

Simulation-based benchmarking supported the robustness of CN-aware modeling by demonstrating separation of cancer biology-driven and CN-driven expression effects across diverse signal regimes. These results complement the TCGA analyses and help explain the improved interpretability observed in real cancer datasets.

While the main contribution of our approach is to include CNVs in the DEGs picture, DeConveil still has some limitations. For instance, we assumed a linear modelling framework that may not fully capture nonlinear relationships between CNVs and gene expression. These are particularly important for high-ploidy states and RNA buffering effects that saturate the signal [4, 14]. In this direction, models with diminishing returns might be considered to improve our approach.

A related challenge arises from limited statistical power in DE testing, which can lead to false negatives and ambiguous gene-dosage classification. In this context, tumor-only CN-expression regression provides a complementary perspective by directly quantifying dosage-expression coupling within tumors but lacks the ability to distinguish DIGs in the absence of normal controls. DeConveil’s joint tumor-normal modeling framework addresses this limitation by enabling separation of biology-driven regulation from CN-driven effects, while complementary CN-only regression analyses, as demonstrated here, can aid in contextualizing and stratifying gene-dosage classes.

Another key limitation is that DeConveil models CNV effects at the individual gene level without accounting for indirect transcriptional effects. CNVs in transcription factors (TFs) or signaling genes can alter the expression of downstream genes, lacking CNVs [47]. Future extensions could integrate gene regulatory network (GRN) models [47–49] to distinguish direct from indirect CNV effects.

Finally, DeConveil does not explicitly model intra-tumor heterogeneity, assuming a single CN state per gene per sample derived from segmented bulk data. As a result, subclonal CNV variation and its potential impact on gene expression are averaged out. Studying rare subclonal events, particularly in the context of dosage-compensation, may be especially relevant in cases of acquired resistance to therapy. Future versions could integrate single-cell data to improve resolution.

## Materials and methods

### CN-aware differential gene expression modeling

Bulk RNA-seq experiments generate a matrix of read counts $Y\;\in \;R^{N\;\times G}$ that reflect the abundance of *G* transcripts detected across *N* samples. For each gene *g* entry, we model the corresponding count vector $y_{g}$ using a negative binomial distribution [50,51] to account for overdispersion:


$$y_{g\;}\sim \text{ NB}(\mu_{g},\;\theta_{g})$$ (1)

where $y_{g}$ represents the observed read counts for gene *g* across samples, $\mu_{g}\;$ is the expected gene-specific mean expression, $\theta_{g}$ captures variability in expression.

Therefore, the likelihood L of observed read counts for gene *g* is defined as


$$L(y_{g\;}|\;\mu_{g}\;,\;\theta_{g})\;=\;\frac{\Gamma (y_{g}\;+\;\text{1}/\theta_{g})}{\Gamma (y_{g}\;+\;\text{1})\;\Gamma (\text{1}/\theta_{g})}\;(\frac{\text{1}}{\text{1}\;+\;\mu_{g}\;\theta_{g}\;}{)}^{\text{1}/\theta_{g}}\;(\frac{\mu_{g}\;\theta_{g}}{\text{1}\;+\;\mu_{g}\;\theta_{g}\;}{)}^{y_{g}}\;$$ (2)

To model gene expression as a function of covariates, we use a GLM with logarithmic link:


$$log{\;q}_{g,n}\;=\;\sum_{f}x_{n,f\;}\beta_{g,f}\;$$ (3)

where $q_{g,n}\;$ is the expected expression level, $x_{n,f}\;$ represents covariates (e.g., tumor/normal condition), $\beta_{g,f}\;$ are the regression coefficients to be estimated.

We assume that DNA copy number measurements directly influence RNA-seq read counts. For example, a CN of 4 could result in doubled expression compared to a diploid control. Therefore, we modify the baseline GLM to incorporate CNV effects


$$\mu_{g,n\;}=\;s_{n}\;(CN_{g,n}/2)\;\{exp({\text{X}}_{n,f}\;\beta_{g,f}\;)\}\;\;\;\;\;\;\;\;\;\;\;\;\;\;\;\;\;$$ (4)

where $s_{n}\;$ is a sample-specific normalization factor calculated using median-of-ratios method used in DESeq2 [22], while ${CN}_{g,n}/\text{2}\;$ represents a gene and sample specific vector of CN dosage scaling factors. The division by 2 likely normalizes CN values relative to the diploid state (where CN = 2 is the reference). If ${CN}_{g,n}=\text{2}$, this term becomes 1, meaning the expression is unaltered; if ${CN}_{g,n}>\text{2}\;$ (gains/amplifications), this term scales expression up, if ${CN}_{g,n}<\text{2}\;$ (deletions), this term scales expression down. These components adjust the expected mean to account for systematic variables, including CN and sequencing depth.

DeConveil requires three input matrices: matched mRNA read counts $Y\;\in \;R^{N\;\times G}$ and absolute copy number values $CN\;\in \;R^{N\;\times G}$ (for normal diploid samples we assign CN = 2) with rows corresponding to genes and columns to samples, and design matrix $X\;\in \;R^{N\;\times F}$ encoding sample conditions.

The design matrix X is structured as follows: rows correspond to individual samples, and each column represents a feature. In its simplest form, X consists of: intercept column (constant 1 for all samples, modeling baseline expression), condition column (binary indicator: 0 = normal, 1 = tumor). For a dataset with *n* samples and fcovariates, is an n×f  matrix. The model learns an *f-*dimensional coefficient vector, where f  is the number of covariates.

DeConveil fits GLM for each gene and employs an empirical Bayes approach, as in DESeq2 [22]. Initially, the maximum likelihood estimation (MLE) is used to learn $\theta_{g\;}$ and $\beta_{g}$ by maximizing log-likelihood of NB distribution. Subsequently, maximum a posteriori (MAP) estimator, namely approximate posterior estimation for GLM (apeglm) [52] method is used to apply shrinkage to both coefficients. Log₂FC calculation is derived from estimated coefficients ${\;\beta }_{g}$.

The regression ${\;\beta }_{g}$ coefficients for each gene are analyzed using the Wald test [53]. Wald test was applied to test for the statistical significance (p-value) in observed expression differences between tumor and normal sample groups. We evaluate the null hypothesis $H_{\text{0}}:\;c\beta_{\text{0}}\;=\;\text{0}$, where *c* is an *f*-dimensional contrast vector, that selects specific linear combinations of coefficients to test for DE.

### Multiple testing framework

When testing thousands of genes simultaneously, p-values must be adjusted to control false discoveries that arise from multiple comparisons. To integrate evidence from CN-naive and CN-aware analyses, we adopted a gene-level multiple testing framework inspired by stageR [54]. The primary goal of this framework is to reduce inflated error rates caused by applying independent multiple-testing corrections to correlated models, while retaining sensitivity to signals captured by either model.

For each gene *g,* we define two null hypotheses:

$\overset{\left(N\right)}{\underset{\text{0},g}{H}}$: gene *g* is not DE under the CN-naive model,

$\overset{\left(A\right)}{\underset{\text{0},g}{H}}$: gene *g* is not DE under the CN-aware model.

Rather than treating these hypotheses as belonging to separate testing families, we define a gene-level global null hypothesis:


$$\overset{\left(N\right)}{\underset{\text{0},g}{\overset{\left(global\right)}{\underset{\text{0},g}{H}}=H}}\;\cap \;\overset{\left(A\right)}{\underset{\text{0},g}{H}}$$ (5)

which states that gene *g* shows no evidence of DE under either modeling assumption.

To test the global null hypothesis, we compute an omnibus screening [55] p-value by aggregating the unadjusted p-values from the CN-naive and CN-aware analyses using the Simes procedure [56]. Let $\overset{\left(N\right)}{\underset{g}{p}}$ and $\overset{\left(A\right)}{\underset{g}{p}}\;$ denote the unadjusted p-values from the CN-naive and CN-aware tests, respectively. The Simes screening p-value is defined as:


$$\overset{\left(S\right)}{\underset{g}{p}}\;=\;min(\text{2}\;\cdot min(\overset{\left(N\right)}{\underset{g}{p}},\;\overset{\left(A\right)}{\underset{g}{p}}),\;max(\overset{\left(N\right)}{\underset{g}{p}},\;\overset{\left(A\right)}{\underset{g}{p}}))\;$$ (6)

This omnibus test evaluates the global null hypothesis and is sensitive to signals detected by either model.

We then apply Benjamini-Hochberg (BH) [57] method once at the screening stage to the set of $\left\{\overset{\left(S\right)}{\underset{g}{p}}\;\right\}$ across all genes, controlling the FDR at level α. This yields a set *R* of genes that pass screening and are considered to show evidence of DE under at least one modeling assumption. Applying BH only once at the screening stage avoids the pitfalls of performing independent FDR corrections for the CN-naive and CN-aware models, which does not guarantee overall error control.

For genes passing the screening step, we perform a confirmation stage in which the two component hypotheses $\overset{\left(N\right)}{\underset{\text{0},g}{H}}\;$ and $\overset{\left(A\right)}{\underset{\text{0},g}{H}}\;$ are tested conditionally. Within each screened gene, multiplicity is controlled using a Holm procedure as implemented in the stageR framework, thereby controlling the family-wise error rate (FWER) within the gene, conditional on passing screening. This yields confirmed DE calls under each model:


$$\overset{\left(N\right)}{\underset{g}{DE}}\in \;\left\{\text{0},\;\text{1}\right\},\;\overset{\left(A\right)}{\underset{g}{DE}}\in \;\left\{\text{0},\;\text{1}\right\}\;$$ (7)

Therefore, we used for gene-dosage classification based on the joint pattern of confirmed DE decisions together with estimated log₂FC (magnitude of DE).

### Differential expression classification framework

Each gene is evaluated using two DE models:

1. a CN-naive model (PyDESeq2), which does not account for CN effects;
2. a CN-aware model (DeConveil), which incorporates sample- and gene-specific CN in the DE analysis.

A gene *g* in sample *n* is classified as DE if it meets both of the following criteria in a given model: adjusted p-value < 0.05 and absolute log₂FC > 1. This binary DE status (1 = DE, 0 = not-DE) is evaluated independently for each gene under both models, resulting in a two-bit DE status vector per gene:


$${\text{DE}}_{g}\;=\;({\text{DE}}_{naive,\;}\;{\text{DE}}_{aware\;})\;\in \;\left\{\text{0},\;\text{1}\right\}\;$$ (8)

Based on DE status across both models, genes are classified into four mechanistic categories:

1. DSGs: $\overset{\left(N\right)}{\underset{g}{DE}}=\;\text{1}\;$ and $\overset{\left(A\right)}{\underset{g}{DE}}=\text{0}$
2. DIGs: $\overset{\left(N\right)}{\underset{g}{DE}}=\;\text{1}\;$ and $\overset{\left(A\right)}{\underset{g}{DE}}=\;\text{1}$
3. DCGs: $\overset{\left(N\right)}{\underset{g}{DE}}=\;\text{0}\;$ and $\overset{\left(A\right)}{\underset{g}{DE}}=\;\text{1}$
4. Non-DEGs: $\overset{\left(N\right)}{\underset{g}{DE}}=\;\text{0}\;$ and $\overset{\left(A\right)}{\underset{g}{DE}}=\;\text{0}$.

This classification provides a binary framework that helps to disentangle CN-driven transcriptional changes from regulatory expression shifts.

### Real datasets

The data analyzed in this study were sourced from GDC (Genomic Data Commons) Data Portal (https://portal.gdc.cancer.gov/). Specifically, we used aneuploid solid cancer datasets from TCGA, including lung adenocarcinoma (LUAD, 100 samples), breast invasive carcinoma (BRCA, 220 samples), liver hepatocellular carcinoma (LIHC, 102 samples), lung squamous cell carcinoma (LUSC, 100 samples), and kidney renal clear cell carcinoma (KIRC, 146 samples). We downloaded matched primary tumor and normal samples, including absolute gene-level copy number data, mRNA-seq read counts, and clinical information, using the TCGAbiolinks Bioconductor R package (v.2.30.4). The METABRIC DNA CN data and gene expression RNA-seq data were downloaded from the cBioPortal database (https://www.cbioportal.org/).

### Data preprocessing

In RNA-seq data, genes with low expression in normal tissue were filtered out from the analysis (mean expression across samples <10 read counts) to minimize noise. The genes obtained after filtering were used in further differential expression tests. Significant DEGs were identified based on the following criteria: |log₂FC| ≥ 1 and *p* < 0.05.

For the exploratory analysis of the relationship between gene CN and mRNA expression, z-score normalization was applied to the log-transformed mRNA-seq data. For the CN data, the mean CN value for each gene was calculated across samples, and CN states were categorized as follows: 1 (CN mean > 0.0 and ≤ 1.7), 2 (CN mean > 1.7 and ≤ 2.5), 3 (CN mean > 2.5 and ≤ 3.5), 4 (CN mean > 3.5 and ≤ 4.5), and 5 (CN mean > 4.5). Additionally, Principal Component Analysis (PCA) and k-means clustering were applied to select patients for exploratory analysis based on their CN profiles.

### CNV groups definition

For the TCGA datasets used to test DeConveil, CNV groups were defined based on the following criteria: neutral (CN mean > 1.7 and ≤ 2.5), gain (CN mean > 2.5 and ≤ 3.5), and amplification (CN mean > 3.5). Conversely, CN loss was categorized as loss for genes exhibiting CN values 0 or 1 in at least 25% of tumor samples that were considered as frequently deleted.

### Simulation benchmarking

To benchmark DeConveil under controlled conditions, we developed a CN-aware RNA-seq simulation framework. Synthetic RNA-seq counts were generated using a NB model with gene-wise mean expression and dispersion parameters inferred from real TCGA-BRCA dataset to ensure realistic mean–variance relationships. DE between tumor and normal samples was introduced through a combination of simulated biological regulation and somatic CN alterations. Each simulated gene was associated with two levels of ground truth:

(i) a binary DE status (DE/ non-DE), and (ii) a gene-dosage class label capturing the mechanistic relationship between GE and CN. Specifically, genes were assigned to one of four categories: DSGs, DCGs, DIGs, or non-DEGs. These classes served as the reference for classification performance evaluation.

A full description of the generative model, parameter sampling, and CN perturbation strategy is provided in Supplementary S1 Text.

Simulations were performed across various settings, varying sample size per condition (n = 10, 20, 40, 60), CN signal strength (weak and strong), and tumor CN heterogeneity. Each configuration included 5000 genes and 20 replicates to ensure statistical robustness.

### Evaluation of DeConveil’s ability to remove CNV as a confounder

To assess how effectively DeConveil removes CN-driven confounding from DE estimates, we designed simulations with known ground truth, focusing on both DE detection accuracy and effect-size estimation. Each simulated dataset included 10% DSGs, 40% DIGs, and 50% non-DEGs to ensure a balanced test scenario [58]. DeConvel’s performance was compared to CN-naive DE method PyDESeq2, and results from each method were evaluated against the known ground truth.

DE detection performance was evaluated using a confusion-matrix framework, treating DIG genes as true positives and DSG and non-DEG genes as true negatives. Specifically, we defined:

- True Positives (TP): correctly identified DIGs,
- False Positives (FP): DSGs or non-DEGs incorrectly identified as DE,
- True Negatives (TN): non-DEGs or DSGs correctly identified as non-DE.
- False Negatives (FN): DIGs missed by the method.

From these quantities, we calculated standard performance metrics:


$$Precision\;=\;\frac{TP}{TP\;+\;FP}$$ (9)

$$Recall\;=\;\frac{TP}{TP\;+\;FN}$$ (10)

$$F\text{1}-score\;=\;\frac{\text{2}\;\cdot Precision\;\cdot Recall}{Precision\;+\;Recall}$$ (11)

$$MCC\;=\;\frac{TP\cdot TN\;-\;FP\cdot \;FN}{\sqrt{\left(TP\;+\;FP)\cdot (TP\;+\;FN)\;\cdot \;(TN\;+\;FP)\;\cdot \;(TN+FN\right)}}$$ (12)

These metrics quantify each method’s ability to distinguish truly DE genes from CN-driven false positives.

To evaluate effect-size accuracy, we compared the estimated log_2_FCs from each method to the ground-truth biological effects used in the simulations. We quantified accuracy using the mean squared error (MSE) and Pearson correlation between estimated and true log_2_FC values across all genes. Lower MSE and higher correlation indicate more accurate and less biased effect-size estimation.

This analysis isolates the effect-size correction problem and directly assesses whether CN-aware modeling improves interpretability and reduces bias introduced by CN variation.

### Validation of DeConveil classification performance

To evaluate DeConveil’s ability to correctly classify genes into mechanistically distinct gene-dosage categories, we leveraged the simulated ground-truth dosage labels. Each simulated gene was assigned to one of four classes: DSGs, DCGs, DIGs, or non-DEGs.

Gene-dosage classification was derived from the joint outcomes of CN-naive and CN-aware DE analyses. For each gene, DE significance was assessed separately under the CN-naive and CN-aware models, using multiple testing corrections based on the omnibus-based stageR framework. Classification into gene-dosage categories was then determined according to a predefined decision logic that integrates the confirmed DE status under both models.

Classification accuracy was quantified by comparing predicted gene-dosage labels to the known simulator ground truth. Performance was evaluated using the same confusion-matrix–based metrics described above (precision, recall, F1-score, and MCC) to ensure consistency across benchmarking analyses.

This evaluation isolates the gene-dosage classification task from binary DE detection and directly assesses whether explicit CN-aware modeling performs discrimination of distinct dosage behaviors beyond standard DE testing.

### Robustness of DeConveil to CNV estimation noise

To simulate realistic uncertainty in CNV quantification we perturbed 5–20% of randomly selected CN matrix entries with additive discrete noise values. Specifically, we sampled these values from a weighted distribution: [−2 (5%), −1 (30%), 0 (30%), 1 (30%), 2 (5%)]. This weighting scheme reflects common CNV estimation errors, where moderate deviations are more frequent than extreme ones, mimicking variability typically encountered in low-purity or low-coverage tumor samples.

Both RNA-seq counts and CN profiles were simulated by subsampling from real aneuploid TCGA cancer samples, ensuring biologically plausible input patterns.

To quantify robustness, we evaluated:

1. Gene classification stability using the Jaccard index [58, 59], calculated between the sets of genes assigned to each group (DIGs, DSGs, DCGs, and non-DEGs) in the noise-free and noise-perturbed datasets. The index was computed as:

$$J(A,B)\;=\;\frac{\left|A\;\cap \;B\right|}{\left|A\;\cup \;B\right|}\;$$ (13)

where *A* and *B* are gene group memberships before and after noise. Results were averaged across 5 replicates and noise levels. A high Jaccard index (closer to 1) indicates strong agreement and stability of classification.

1. Effect size stability via Pearson correlation (R²) between estimated and true log2 fold changes.
2. Significance stability using Spearman correlation (R²) of adjusted p-value rankings (FDR)

All metrics were computed across 5 simulation replicates and sample sizes (n = 10, 20, 40, and 60).

### Functional enrichment analysis

Overrepresentation analysis of DE gene categories identified by DeConveil was performed using the Gene Ontology database provided by clusterProfiler (v.4.2.2) [60] R/Bioconductor package. For each gene category (DSGs, DIGs, and DCGs) we performed a hypergeometric test using the function *enrichGO*. We focused on biological process (BP) GO terms, filtering for gene sets with sizes between 10 and 350 genes. A p-value cutoff of 0.05 was used as the significance threshold for GO term identification.

### Survival analysis

We used gene groups identified by DeConveil to identify prognostic gene signatures associated with patient survival outcomes. Log-normalized RNA-seq expression data from each gene category (DSGs, DIGs, and DCGs) were scaled by corresponding CN values to account for gene dosage effects. These expression data were then integrated with clinical time-to-event data for survival modeling.

As a first step, we applied the Cox proportional hazards model using the survival R package (v.3.8-3) to identify genes significantly associated with overall survival. Genes with an adjusted p-value < 0.05 were retained for further analysis. To reduce dimensionality and select the most predictive features, we applied LASSO-penalized Cox regression [61] using the glmnet R package (v.4.1-8). Genes with non-zero LASSO coefficients were selected to form the final prognostic gene signature. A prognostic score was then computed for each patient as a weighted linear combination of the expression levels of these selected genes, using their corresponding LASSO-derived coefficients. Based on the median prognostic score, patients were stratified into high-risk (score > median) and low-risk (score ≤ median) groups.

To assess the survival differences between these groups, we generated Kaplan–Meier survival curves and performed log-rank tests. A p-value < 0.05 was considered statistically significant.

For comparative analysis, we also evaluated DE genes identified using a CN-naive model, where gene expression was not adjusted for CN effects. These DE genes were subjected to the same survival analysis pipeline.

For external validation of the model’s predictive performance, we used the METABRIC dataset (n = 520 patients). The previously derived LASSO-selected gene signature was applied to this independent cohort, and prognostic scores were calculated accordingly. Model performance was evaluated using the concordance index (C-index), which quantifies the concordance between predicted risk scores and observed survival outcomes.

## Supporting information

S1 Fig Boxplots show the relationship between mRNA Z-score and CN groups across tumor types (BRCA, LIHC, LUSC). (TIFF)

S2 Fig Statistical calibration and classification performance under CN-aware simulation. **(A)** Null p-value distributions under CN-aware (top) and CN-naive (bottom) models across increasing sample sizes per condition (10, 20, 40, 60), show approximately uniform behavior under the global null. Dashed lines indicate the expected Uniform (0,1) density. (**B)** Empirical cumulative distribution functions (ECDFs) of null p-values compared with the Uniform (0,1) reference, stratified by sample size, demonstrate well-calibrated type I error control for both CN-aware and CN-naive models. (**C)** Confusion matrices for gene-class classification using the Simes omnibus plus stageR FDR control strategy, shown for DCGs, DSGs, and DIGs versus all other genes under strong CN signal at sample sizes (n = 20, 60). (**D)** Corresponding confusion matrices for a standard analysis pipeline applying Benjamini-Hochberg correction separately per model. (**E)** Precision, recall, and F1-score for DCG, DSG, and DIG classification as a function of sample size, comparing the stageR-based pipeline with the standard pipeline. (**F)** Sankey diagram summarizing gene-class assignments under the standard pipeline and the stageR pipeline for S = 60, strong CN signal, and G = 5000 genes, illustrating differences in classification outcomes across DCG, DIG, DSG, and non-DEG groups.
(TIFF)

S3 Fig Robustness of DeConveil to CNV estimation noise across gene groups and sample sizes. Simulated CN values were randomly perturbed in 5%, 10%, 15%, or 20% of the gene-sample matrix using additive discrete noise drawn from [−2, 2]. Panels show metrics across 5 replicates per condition. (**A)** Mean Jaccard index across replicates assessing gene group classification stability. (**B)** Pearson R2 correlation between noise-free and noisy log_2_FC. (**C)** Spearman R2 correlation between FDR values under noise-free vs. noisy conditions. DeConveil is resilient to CN noise, especially in DIG and non-DEG groups, while CN-sensitive groups (DSG, DCG) show moderate declines at higher noise levels and larger sample sizes.
(TIFF)

S4 Fig Gene classification transitions under single versus independent BH correction across TCGA cancer types. Sankey diagrams illustrate gene classification transitions between a standard pipeline with independent BH corrections and the omnibus-stageR-based pipeline with a single BH correction across multiple TCGA cancer cohorts. Flows indicate reclassification of genes among DCG, DIG, DSG, and non-DEG groups, with numbers showing gene counts per category.
(TIFF)

S5 Fig Impact of CN corrections on DGE analysis across aneuploid cancer types (LUSC, BRCA, LIHC, KIRC). **(A)** Distribution of CN states across tumor samples. (**B)** Gene classification by CN status (amplification, gain, loss, neutral) and gene group (DIGs, DSGs, DCGs); stacked bars show proportions, with percentages indicating each group’s contribution to the total gene set. **(C)** Volcano plots comparing PyDESeq2 and DeConveil DGE results (threshold for significant DE: |log₂FC| > 1 and FDR < 0.05)**. (D)** Sankey diagram showing shifts in gene expression classification (upregulated, downregulated, non-significant (n.s)) between PyDESeq2 and DeConveil following CN correction.
(TIFF)

S6 Fig Impact of CN corrections on DGE analysis across aneuploid cancer types (LUSC, BRCA, LIHC, KIRC). **(A)** Comparison of effect size (log_2_FC) between PyDESeq2 and DeConveil models across different CN states (loss, neutral, gain, and amplification). The diagonal reference line represents a one-to-one correlation; deviations from this line indicate differences in effect size or FDR between the two approaches. (**B)** Comparison of FDR between PyDESeq2 and DeConveil models across different CN states. (**C)** Distribution of effect size differences (log₂ scale) across CN states.
(TIFF)

S7 Fig Gene Ontology (GO) term enrichment analysis for biological processes associated with DSGs, DIGs, and DCGs across LUAD, LUSC, and BRCA. The dot plots represent significantly enriched biological processes for each gene category. The size of the dots corresponds to the number of genes associated with the process, while the color represents the statistical significance of enrichment (-log₁₀ adjusted p-value).
(TIFF)

S8 Fig Agreement between DeConveil and tumor-only NB regression gene-dosage classifications. **(A)** Concordance between DeConveil and NB regression gene classes under moderate (τ > 0.3) and strong (τ > 1.0) effect-size thresholds. Heatmaps show row-normalized proportions of DeConveil classes (DSG, DIG, DCG) within each NB regression class (DSG, DCG, HYPER, OTHER). **(B)** Reassignment of DeConveil DCGs to NB regression classes under moderate and strong effect-size thresholds. Stacked bars indicate the proportion of DCGs assigned to each NB class. (**C)** Normalized entropy of NB class assignment probabilities for DCG subtypes (pure, directional, ambiguous) under moderate and strong effect-size thresholds. Dashed lines indicate entropy cutoffs used for DCG subtype stratification.
(TIFF)

S9 Fig Representative expression-CN relationships for gene-dosage classes in LUAD. Scatter plots show normalized gene expression as a function CN for representative genes from each class: DSG, DCG and DIG (identified by DeConveil), and hyperactivated gene (HYPER) identified by NB regression. Solid blue lines indicate observed linear trends fitted to tumor samples, while dashed orange lines represent the expected CN-proportional expression. These examples illustrate characteristic dosage-response behaviors associated with each gene class.
(TIFF)

S10 Fig Volcano plots showing prognostic genes identified with Cox/LASSO regression within each gene category (DSGs, DIGs, DCGs). (TIFF)

S1 Table Summary of private gene distribution (%) across gene categories (DSGs, DIGs, and DCGs) and cancer types (LUAD, LUSC, BRCA). This table provides insights into the relative distribution of private genes within each gene category and cancer type, and their overall background proportions.
(XLSX)

S2 Table Summary of gene categories across three cancer types (LUAD, LUSC, BRCA). This table provides the number of genes in three different gene categories (DSGs, DIGs, and DCGs). The table includes the mean number of genes per category, their proportion in percentage, and the number and proportion of shared genes among the categories.
(XLSX)

S1 Text Additional details on simulations. Description of the copy-number–aware RNA-seq simulation framework used in this study.
(PDF)

S2 Text Complementary negative binomial regression model. Details of the complementary negative binomial regression model, implementation, and interpretation of results.
(PDF)
