# Supplementary figures and images for: Extending differential gene expression testing to handle genome aneuploidy in cancer
Source: PLoS Comput Biol. 2026 Mar 27;22(3):e1014134. doi: 10.1371/journal.pcbi.1014134 (PMC13061324; doi:10.1371/journal.pcbi.1014134)

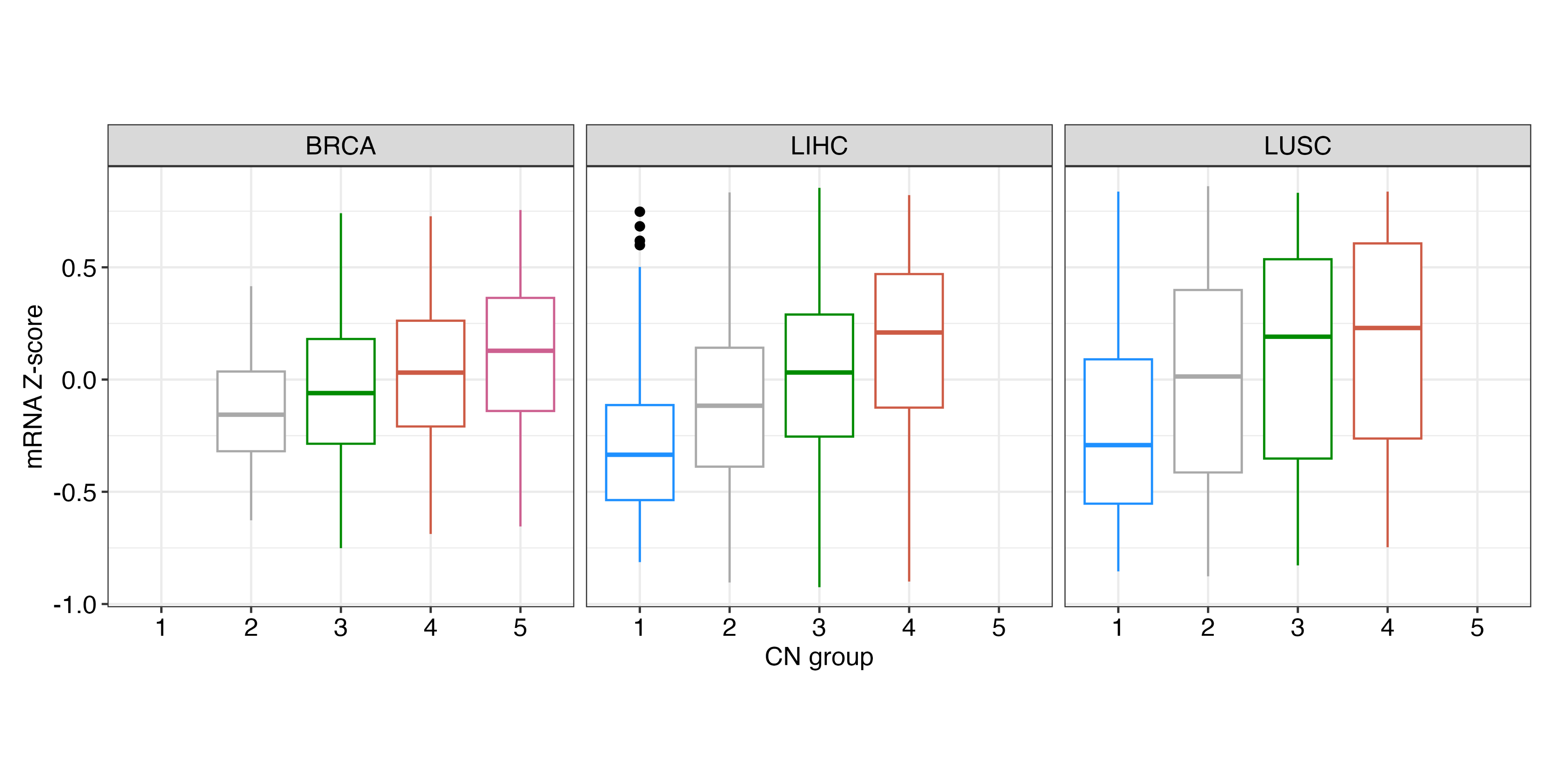

Supplement: S1 Fig — (TIFF) [file pcbi.1014134.s001.tiff]

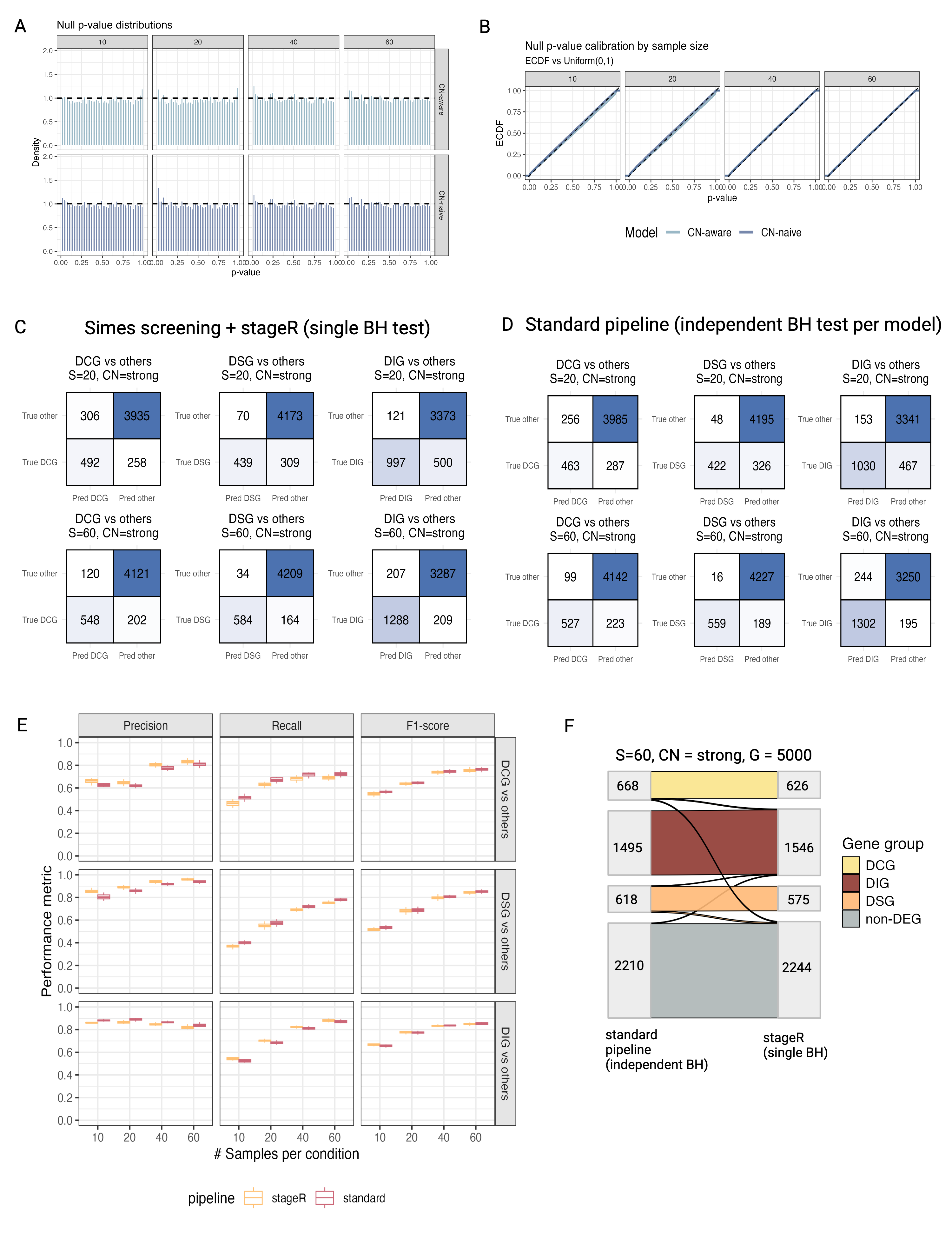

Supplement: S2 Fig — (A) Null p-value distributions under CN-aware (top) and CN-naive (bottom) models across increasing sample sizes per condition (10, 20, 40, 60), show approximately uniform behavior under the global null. Dashed lines indicate the expected Uniform (0,1) density. (B) Empirical cumulative distribution functions (ECDFs) of null p-values compared with the Uniform (0,1) reference, stratified by sample size, demonstrate well-calibrated type I error control for both CN-aware and CN-naive models. (C) Confusion matrices for gene-class classification using the Simes omnibus plus stageR FDR control strategy, shown for DCGs, DSGs, and DIGs versus all other genes under strong CN signal at sample sizes (n = 20, 60). (D) Corresponding confusion matrices for a standard analysis pipeline applying Benjamini-Hochberg correction separately per model. (E) Precision, recall, and F1-score for DCG, DSG, and DIG classification as a function of sample size, comparing the stageR-based pipeline with the standard pipeline. (F) Sankey diagram summarizing gene-class assignments under the standard pipeline and the stageR pipeline for S = 60, strong CN signal, and G = 5000 genes, illustrating differences in classification outcomes across DCG, DIG, DSG, and non-DEG groups. (TIFF) [file pcbi.1014134.s002.tiff]

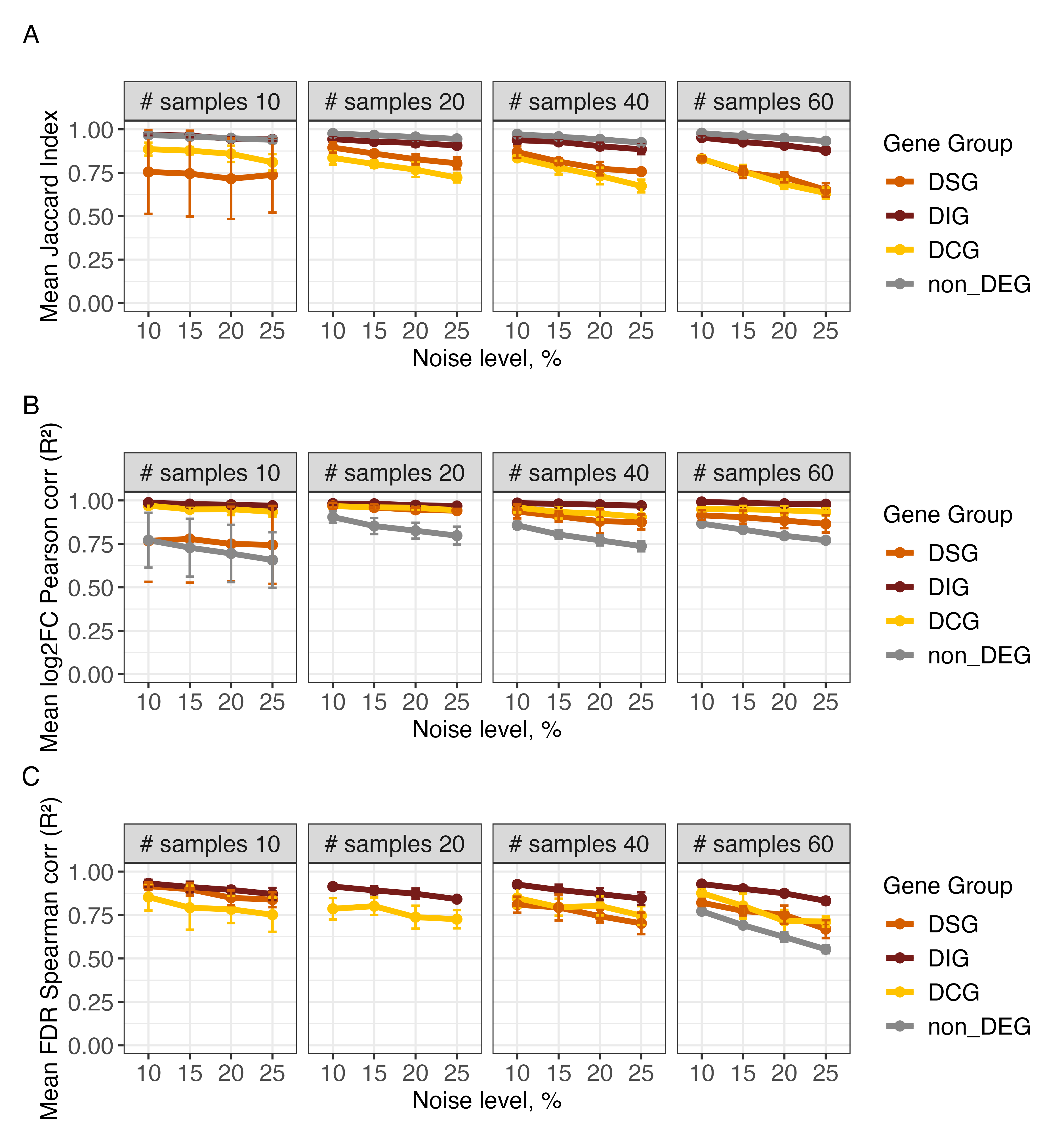

Supplement: S3 Fig — Simulated CN values were randomly perturbed in 5%, 10%, 15%, or 20% of the gene-sample matrix using additive discrete noise drawn from [−2, 2]. Panels show metrics across 5 replicates per condition. (A) Mean Jaccard index across replicates assessing gene group classification stability. (B) Pearson R2 correlation between noise-free and noisy log2FC. (C) Spearman R2 correlation between FDR values under noise-free vs. noisy conditions. DeConveil is resilient to CN noise, especially in DIG and non-DEG groups, while CN-sensitive groups (DSG, DCG) show moderate declines at higher noise levels and larger sample sizes. (TIFF) [file pcbi.1014134.s003.tiff]

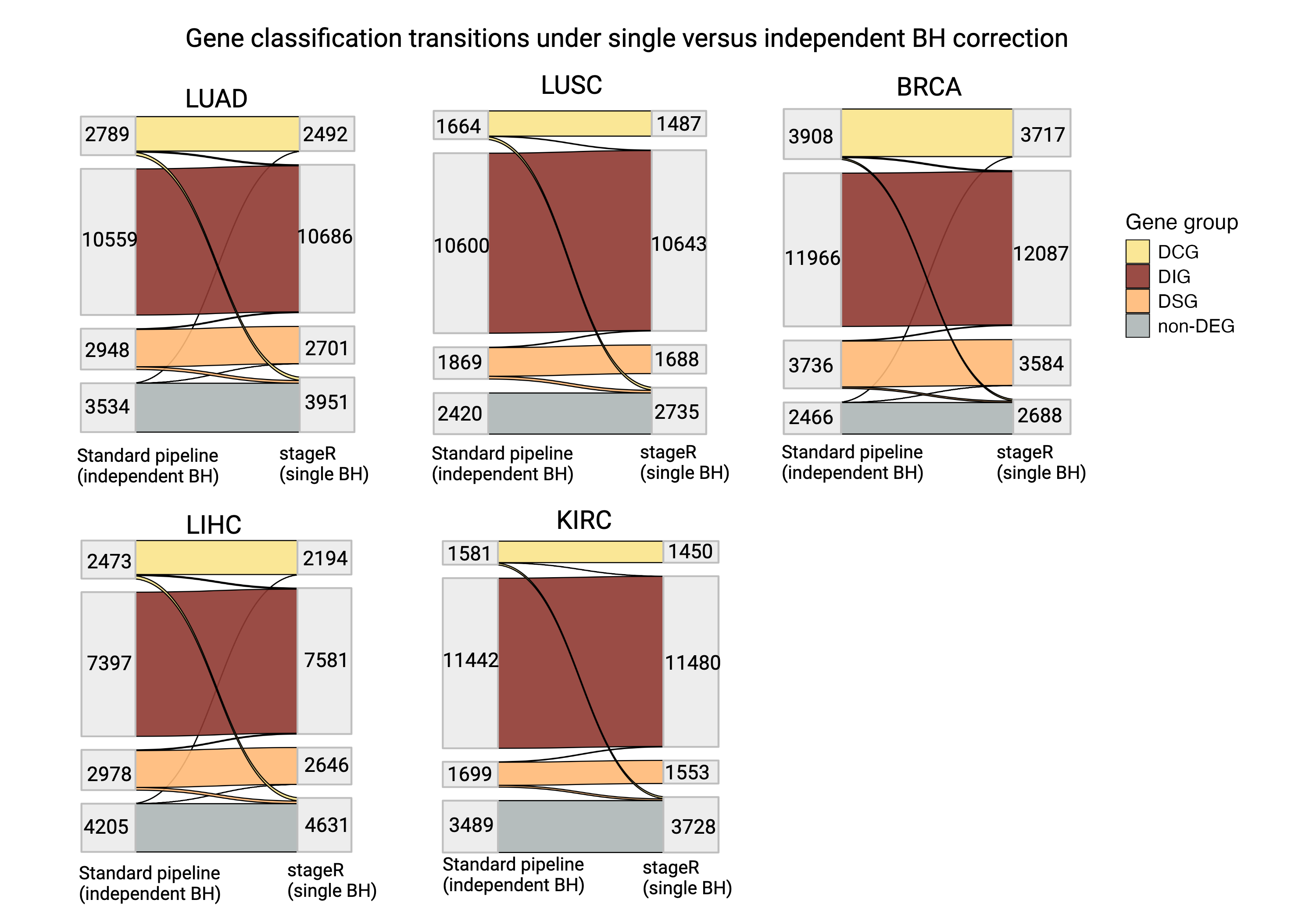

Supplement: S4 Fig — Sankey diagrams illustrate gene classification transitions between a standard pipeline with independent BH corrections and the omnibus-stageR-based pipeline with a single BH correction across multiple TCGA cancer cohorts. Flows indicate reclassification of genes among DCG, DIG, DSG, and non-DEG groups, with numbers showing gene counts per category. (TIFF) [file pcbi.1014134.s004.tiff]

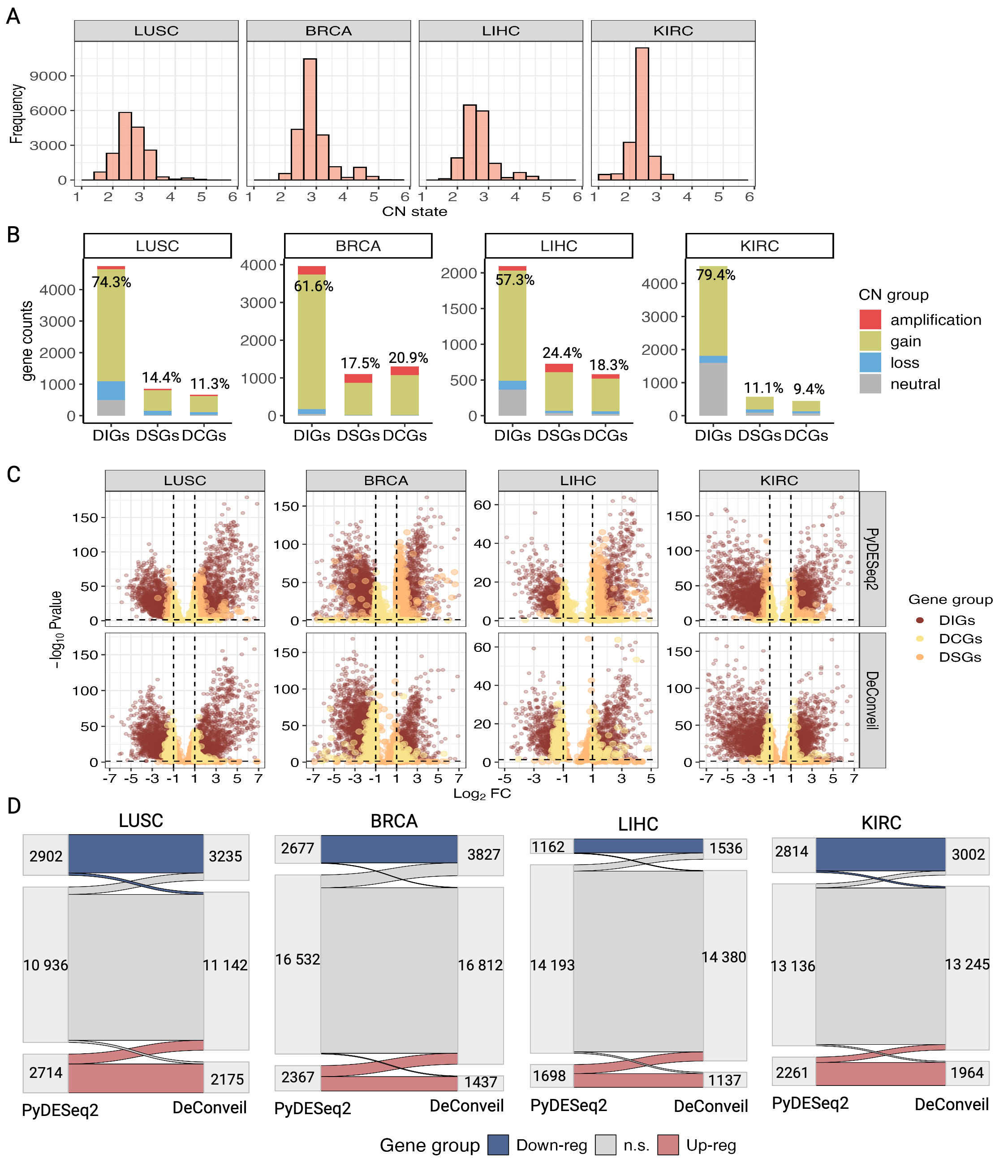

Supplement: S5 Fig — (A) Distribution of CN states across tumor samples. (B) Gene classification by CN status (amplification, gain, loss, neutral) and gene group (DIGs, DSGs, DCGs); stacked bars show proportions, with percentages indicating each group’s contribution to the total gene set. (C) Volcano plots comparing PyDESeq2 and DeConveil DGE results (threshold for significant DE: |log₂FC| > 1 and FDR < 0.05). (D) Sankey diagram showing shifts in gene expression classification (upregulated, downregulated, non-significant (n.s)) between PyDESeq2 and DeConveil following CN correction. (TIFF) [file pcbi.1014134.s005.tiff]

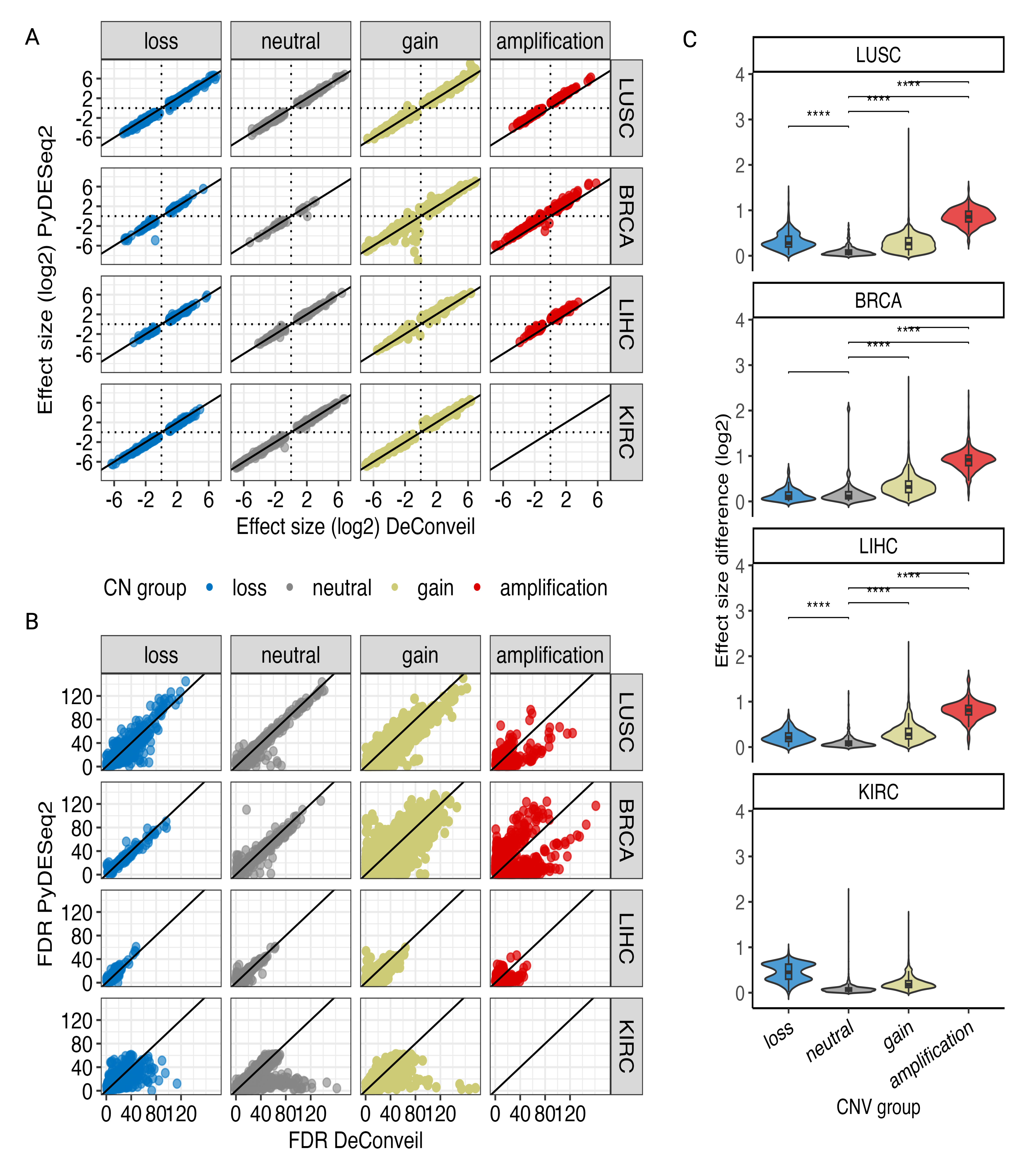

Supplement: S6 Fig — (A) Comparison of effect size (log2FC) between PyDESeq2 and DeConveil models across different CN states (loss, neutral, gain, and amplification). The diagonal reference line represents a one-to-one correlation; deviations from this line indicate differences in effect size or FDR between the two approaches. (B) Comparison of FDR between PyDESeq2 and DeConveil models across different CN states. (C) Distribution of effect size differences (log₂ scale) across CN states. (TIFF) [file pcbi.1014134.s006.tiff]

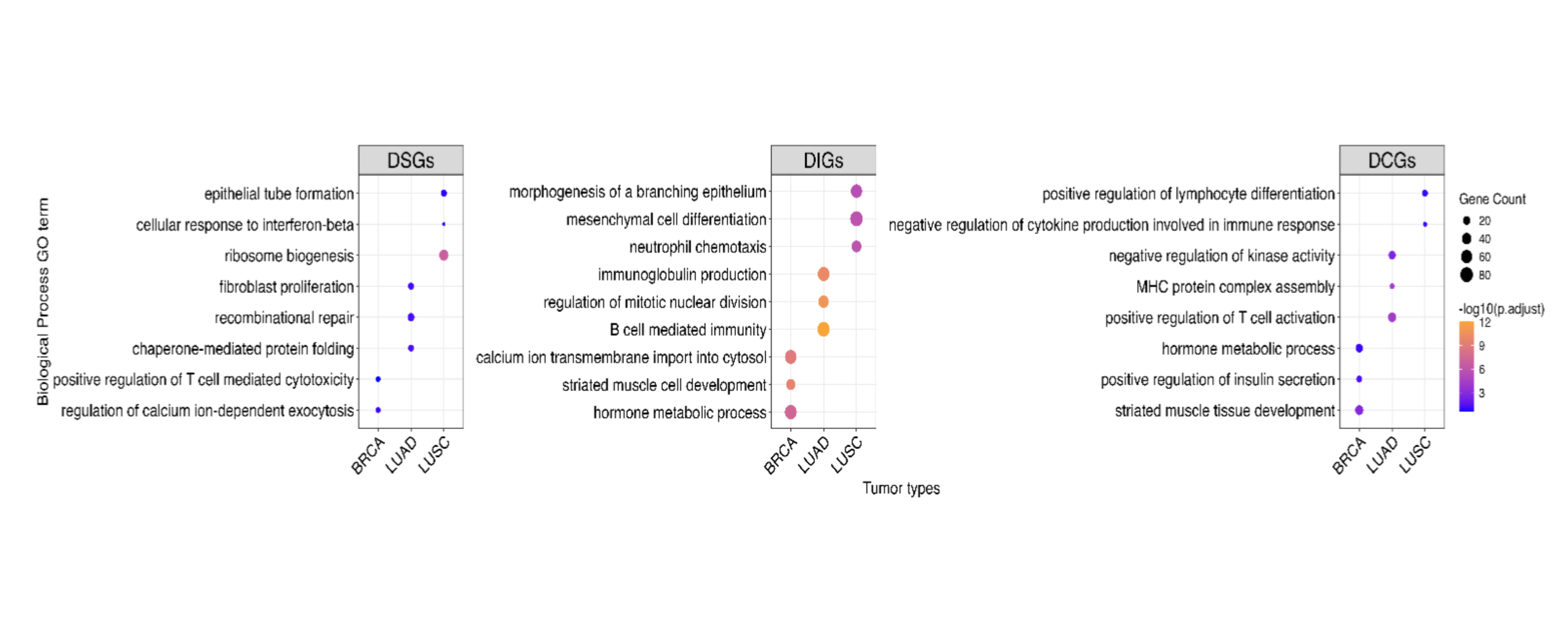

Supplement: S7 Fig — The dot plots represent significantly enriched biological processes for each gene category. The size of the dots corresponds to the number of genes associated with the process, while the color represents the statistical significance of enrichment (-log₁₀ adjusted p-value). (TIFF) [file pcbi.1014134.s007.tiff]

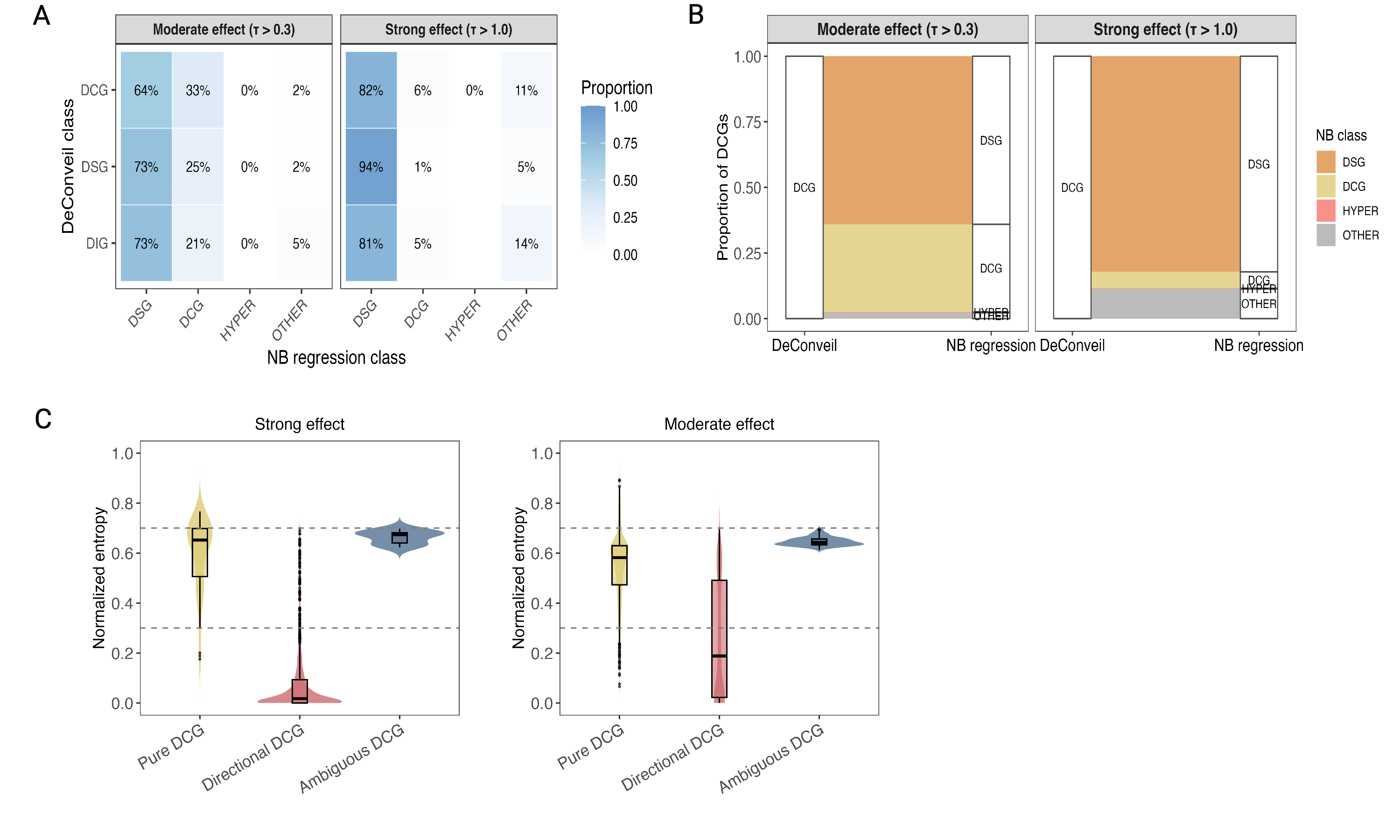

Supplement: S8 Fig — (A) Concordance between DeConveil and NB regression gene classes under moderate (τ > 0.3) and strong (τ > 1.0) effect-size thresholds. Heatmaps show row-normalized proportions of DeConveil classes (DSG, DIG, DCG) within each NB regression class (DSG, DCG, HYPER, OTHER). (B) Reassignment of DeConveil DCGs to NB regression classes under moderate and strong effect-size thresholds. Stacked bars indicate the proportion of DCGs assigned to each NB class. (C) Normalized entropy of NB class assignment probabilities for DCG subtypes (pure, directional, ambiguous) under moderate and strong effect-size thresholds. Dashed lines indicate entropy cutoffs used for DCG subtype stratification. (TIFF) [file pcbi.1014134.s008.tiff]

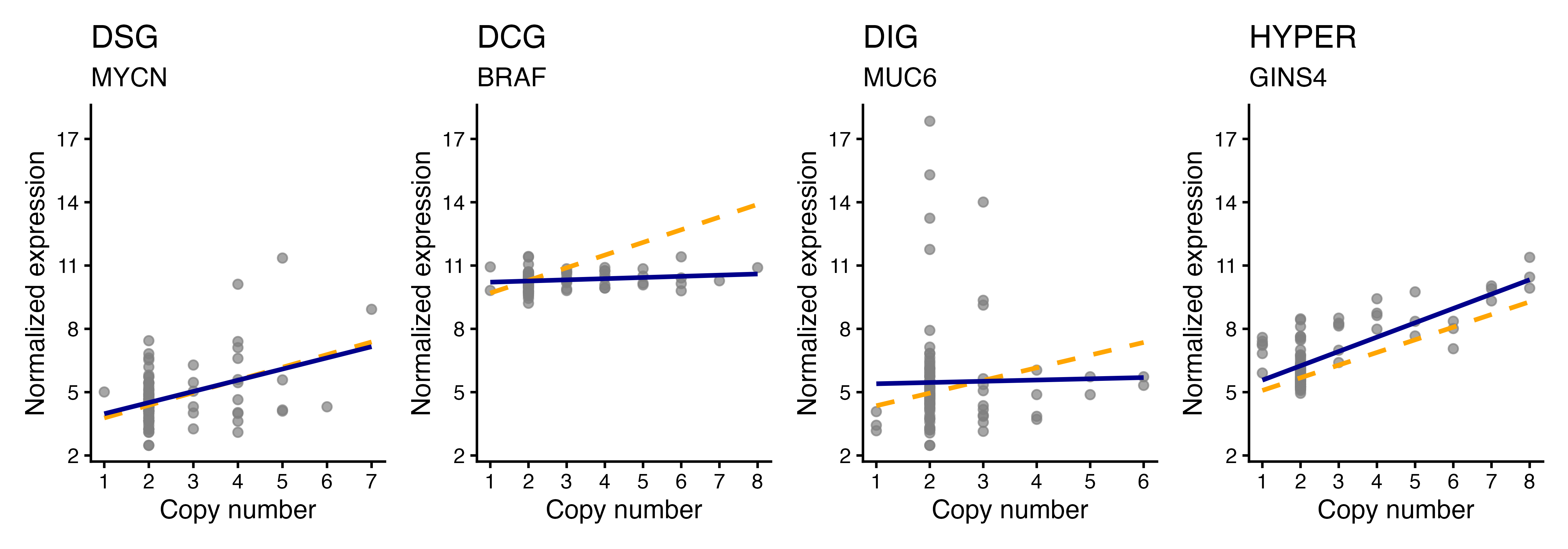

Supplement: S9 Fig — Scatter plots show normalized gene expression as a function CN for representative genes from each class: DSG, DCG and DIG (identified by DeConveil), and hyperactivated gene (HYPER) identified by NB regression. Solid blue lines indicate observed linear trends fitted to tumor samples, while dashed orange lines represent the expected CN-proportional expression. These examples illustrate characteristic dosage-response behaviors associated with each gene class. (TIFF) [file pcbi.1014134.s009.tiff]

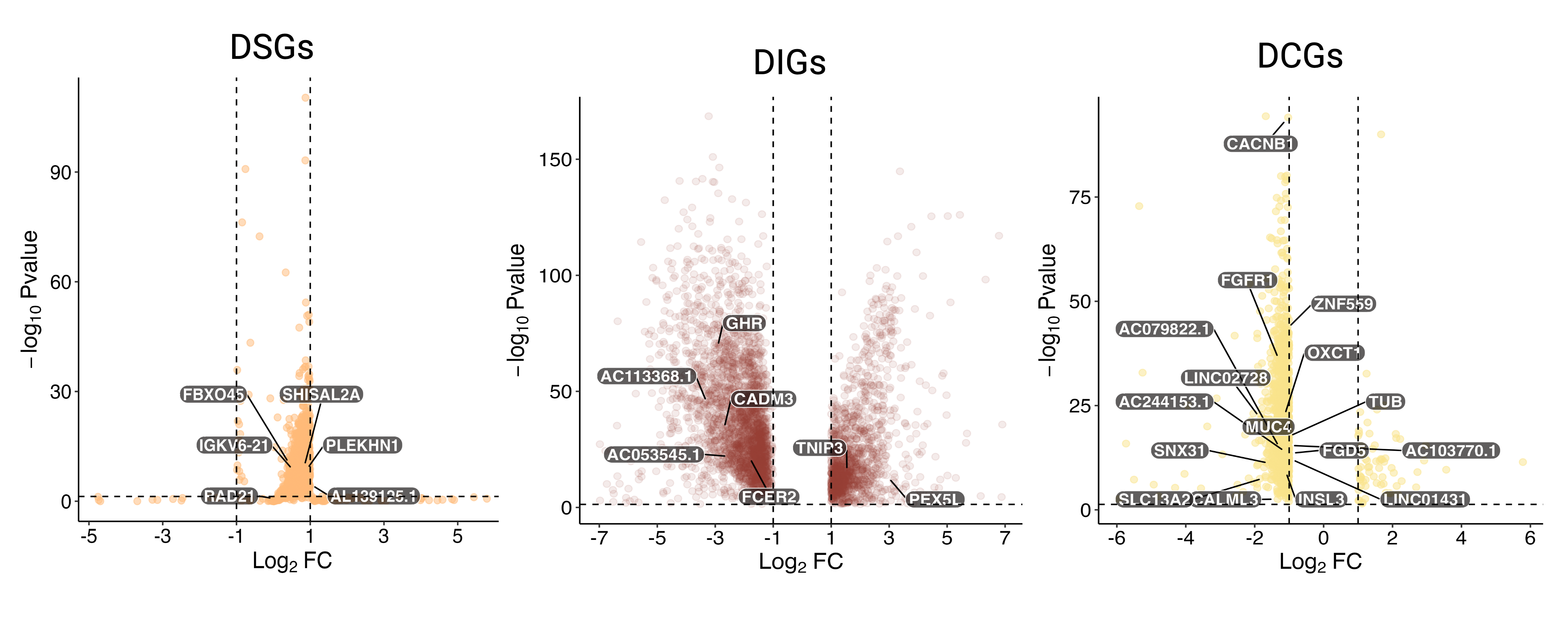

Supplement: S10 Fig — (TIFF) [file pcbi.1014134.s010.tiff]
