# Supplementary material for: Extending differential gene expression testing to handle genome aneuploidy in cancer
Source: PLoS Comput Biol. 2026 Mar 27;22(3):e1014134. doi: 10.1371/journal.pcbi.1014134 (PMC13061324; doi:10.1371/journal.pcbi.1014134)
Supplement: S1 Text — Description of the copy-number–aware RNA-seq simulation framework used in this study. (PDF) [file pcbi.1014134.s013.pdf]

S1 text
Additional details on simulations

Copy-number-aware RNA-seq simulation framework

We designed a copy-number-aware (CN-aware) RNA-seq simulation framework to benchmark `DeConveil` under controlled but biologically realistic conditions. The simulator generates synthetic RNA-seq count data in which differential expression (DE) arises from a combination of DNA CN dosage effects and biology-driven transcriptional regulation.
By varying the strength and interaction of these components, the simulator generates four biologically meaningful gene classes: DIGs, DSGs, DCGs and non-DEGs.
The framework consists of (i) a Negative Binomial (NB) generative model for RNA-seq counts, (ii) parameter inference from real RNA-seq data, (iii) CN profile preprocessing, (iv) assignment of genes to CN-driven dosage classes, and (v) ground-truth DE definitions under CN-naive and CN-aware models.

Generative model

Let *g = 1,...,G* index genes and *n=1,...,N* index samples. Samples are partitioned into normal ($X_n = 0$) and tumor ($X_n = 1$) conditions. Observed RNA-seq counts $\mathbf{Y}_{g,n}$ follow a NB distribution parameterized by a mean $\mu_{g,n}$ and gene-specific dispersion parameter θ_g:

$$\mathrm{Y}_{g,n} \sim \mathrm{NB}(\mu_{g,n}, \theta_g), \qquad \mathrm{Var}(Y_{g,n}) = \mu_{g,n} + \theta_g \mu_{g,n}^2 \qquad (1)$$

Dispersion is constant across samples but varies by gene.
The expected mean expression is modeled on the log scale as:

$$\log(\mu_{g,n}) = \log(\beta_{0,g}) + \beta_{1,g}\, X_n + \log(\frac{CN_{g,n}}{2} + \epsilon) \qquad (2)$$

where

$X_n \in \{0, 1\}$ is the condition indicator (0 = normal, 1 = tumor),
$\beta_{0,g} =$ baseline expression level in normal samples,
$\beta_{1,g} =$ tumor-normal biological effect size,
$\mathrm{C}N_{g,n} =$ integer copy-number for gene *g* in sample *i.* The division by 2 centers CN dosage relative to diploid CN=2 (baseline dosage=1),
$\epsilon = 0.1$: small stability offset.
This formulation ensures that CN effects act multiplicatively on expression and are centered at diploid dosage.

Simulation pipeline

Parameter inference from real RNA-seq data

To preserve realistic mean–dispersion relationships, baseline expression levels and dispersions are estimated directly from real RNA-seq data. Using DESeq2, we fit a simple design (e.g., ~ 1 or ~ condition) to an empirical count matrix and extract:

$$\mu_{0,g} = \frac{1}{N}\sum_{n=1}^{S} \hat{\mu}_{g,n}^{(\mathrm{DESeq2})}, \qquad \theta_g = \hat{\theta}_g^{(\mathrm{DESeq2})} \qquad (3)$$

These empirical estimates are used as parameters in downstream simulations.

Tumor CN homogenization

To avoid excessive CN heterogeneity that may dominate biological effects, the tumor CN matrix can be optionally homogenized prior to simulation while preserving integer CN states. Let f_{dip} and f_{del} denote user-defined proportions of genes subjected to diploid and deletion homogenization, respectively. The homogenization procedure is performed in the following way:

1) diploid enrichment: a random fraction f_{dip} of genes forced to CN = 2 across all tumor samples;
2) deletion enrichment: a fraction of genes f_{del} are forced to CN=1 in tumor to mimic hemizygous deletions commonly observed in cancer.

This preprocessing step enables controlled manipulation of CN signal strength.

Sampling of gene-level effects and dosage class assignment

To generate realistic effect size for log2-fold change (log2FC) under both CN-naive and CN-aware modeling, each gene is first assigned to a dosage class and paired with effect parameters drawn from constrained distributions.
For each gene $g \in \{1, \ldots, G\}$, we sample:
τ_g: biology-driven effect (log scale),
c_g: copy-number–derived expression shift estimated from tumor CN profile, defined as:

$$c_g = \log_2\left(\frac{\tilde{CN}_g}{2} + 0.1\right) \qquad (4)$$

where $\tilde{CN}_g$ is the mean tumor CN for gene *g*,
$\beta_{1,g}$: effect size (log2FC), set to $\beta_{1,g} = \log(2) \cdot \tau_g$.
To explain how strongly gene expression is expected to be driven by CN alterations, we compute the following statistics across tumor samples for each gene *g*:

$$\mu_{CN,g} = \frac{1}{N}\sum_n CN_{g,n} \qquad (5)$$

$$\mathrm{sd}_{CN,g} = \sqrt{\frac{1}{N}\sum_n \left(CN_{g,n} - \mu_{CN,g}\right)^2} \qquad (6)$$

$$\text{efrac}_g = \frac{1}{N}\sum_n \mathbf{1}(CN_{g,n} \neq 2) \qquad (7)$$

These capture CN mean shift, CN heterogeneity, and CN penetrance (the proportion of samples *n* with any deviation from diploid), respectively.
We compute then a smooth CN activity score:

$$\text{score}_g = w_{\text{sd}}\,\text{sd}_{CN,g} + w_{\text{mean}}\,|\mu_{CN,g} - 2| + w_{\text{efrac}}\,\text{efrac}_g \qquad (8)$$

where w_{sd}, w_{mean}, w_{efrac} allow tuning the relative importance of each component.
Genes are ranked by score_g and partitioned into fixed fractions to define CN-driven dosage classes.

Table 1. Simulated gene categories with corresponding CN signals and biological Interpretation.

Fraction	Class	CN signal	Interpretation
40%	non-DEG	none	diploid, stable, no DE
30%	DIG	mild	biology-driven DE
15%	DSG	strong	CN-driven DE
15%	DCG	strong	amplified/deleted at the DNA level but compensated at RNA level

This quantile-based assignment avoids arbitrary CN thresholds and ensures well-controlled CN signal simulation scenarios.
Effect sizes are sampled as follows:

- DIG: strong biological effect with mild CN bias
  $\tau_g \sim U(\pm 0.8,\ \pm 2.5)$
- DSG: expression dominated by CN dosage
  $\tau_g \sim N(0.0,\ 0.1)$
- DCG: genes exhibit strong CN alterations but maintain stable expression through compensatory regulation
  $\tau_g = -c_g + \epsilon_g,\ \epsilon_g \sim N(0.0,\ 0.1)$
  with the constraint $|\tau_g| \geq \delta$, where $\delta = 0.6$ (weak signal) or $\delta = 1.2$ (strong signal) to ensure detectability in CN-aware models.
- non-DEG genes: no biological DE
  $\tau_g \sim N(0.0,\ 0.05)$

Sequencing depth and dispersion perturbation

To mimic variability in sample-specific sequencing depth, each sample *n* is assigned a multiplicative scaling factor:
$s_n \sim U(0.7,\ 1.4)$

If $\mu_{g,n}$ is the expected expression for gene *g* in sample *n,* the depth-adjusted mean becomes: $\mu_{g,n} = s_n \mu_{g,n}$.
To mimic increased tumor heterogeneity, a subset of genes undergoes dispersion inflation in tumor samples

$$\theta_g^{(\text{tum})} = \theta_g^{(\text{tum})} \cdot d_g, \qquad d_g \sim \text{Uniform}(0.5,\ 2.0) \qquad (10)$$

Count sampling

Counts are generated using an equivalent NB parameterization in terms of success probability *p* and size $1/theta$, which is equivalent to the mean–dispersion form in Eq. (1):

$$Y_{g,n} \sim \text{NB}\left(p_{g,n}, \frac{1}{\theta_{g,n}}\right), p_{g,n} = \frac{1/\theta_{g,n}}{1/\theta_{g,n} + \mu_{g,n}} \qquad (11)$$

Normal and tumor samples are concatenated into a single count matrix.

Ground-truth definition and benchmarking labels

DE ground truth corresponds to the biology-driven effect parameter τ_g, which corresponds to the regression coefficient $\beta_{g,1}$ of the CN-aware model.
Under CN-naive model, which do not adjust for CN, the effective tumor–normal $\log_2$FC is:

$$\text{truth}_g^{\text{naive}} = \tau_g + c_g, \qquad c_g = \log_2\left(\frac{\overline{CN}_g^{\text{tumor}}}{2} + 0.1\right) \qquad (12)$$

Because the naive model absorbs CN effects into the regression coefficient, the true naive log2FC is defined as:

$$\text{truth}_g^{\text{naive}} = \tau_g + c_g, \qquad c_g = \log_2\left(\frac{\overline{CN}_g^{\text{tumor}}}{2} + 0.1\right) \qquad (13)$$

This construction yields distinct DE regimes:

- DSGs: c_g dominates ($\tau_g \approx 0$),
- DCGs: $\tau_g \approx -c_g$ (CN effects are compensated, yielding weak/no CN-naive DE but strong CN-aware DE),
- DIGs: τ_g dominates (DE is detected by both CN-naive and CN-aware models),
- non-DEGs: no DE; $\tau_g \approx 0$ and $c_g \approx 0$.

Genes are labeled as truly DE based solely on the magnitude of the biological effect:

$$DE_{truth_g} = \begin{cases} \text{True}, & |\tau_g| > \varepsilon_{\text{truth}}, \\ \text{False}, & \text{otherwise} \end{cases} \qquad (14)$$

with default $\epsilon = 0.25$. In null simulations, all genes are explicitly set to: $DE_{truth_g} = False$
.
