# Supplementary material for: Extending differential gene expression testing to handle genome aneuploidy in cancer
Source: PLoS Comput Biol. 2026 Mar 27;22(3):e1014134. doi: 10.1371/journal.pcbi.1014134 (PMC13061324; doi:10.1371/journal.pcbi.1014134)
Supplement: S2 Text — Details of the complementary negative binomial regression model, implementation, and interpretation of results. (PDF) [file pcbi.1014134.s014.pdf]

S2 Text

Complementary Negative Binomial regression

To provide independent support for `DeConveil's` gene-dosage classifications, we additionally implemented a gene-wise Negative Binomial (NB) regression model [1,2] that associates RNA-seq counts with CN variation across tumor samples. This tumor-only, condition-agnostic framework quantifies copy number (CN)-gene expression (GE) coupling independently of tumor–normal contrasts. This NB regression analysis was used solely as a complementary approach to contextualize and assess agreement with `DeConveil` gene classes.

Data preprocessing

To focus on informative loci, we restricted the analysis to genes exhibiting CN alterations, defined as gains or losses present in at least 30% of samples within each TCGA cohort.

For each gene *g* and sample *n*, integer CN values were decomposed into two components capturing expected dosage scaling and deviation from diploid dosage:

$$c_{g,n} = \frac{CN_{g,n}}{2}, \quad d_{g,n} = \frac{CN_{g,n} - 2}{2} \qquad (1)$$

$c_{g,n}$ represent expected expression scaling relative to diploid state, and $d_{g,n}$ captures expression changes that deviate from dosage expectation.

Model specification

For each gene *g* and sample *n*, observed RNA-seq read counts $\mathbf{Y}_{g,n}$ are modeled with NB parametrization:

$$\mathrm{Y}_{g,n} \sim \mathrm{NB}(\mu_{g,n}, \theta_g) \qquad (2)$$

where $\mu_{g,n}$ is the expected mean, and θ_g gene-specific overdispersion parameter.

The expected expression mean is defined using an identity link as:

$$\mu_{g,n} = s_n(\beta_{1,g}\, c_{g,n}\, p_n + \beta_{2,g}\, d_{g,n}\, p_n + \beta_{3,g}\,(1 - p_n) + \epsilon) \qquad (3)$$

where

s_g is library size scaling factor,

p_n is purity term (tumor cell fraction),

$\beta_{1,g}$ quantifies CN-proportional expression scaling (dosage sensitivity),

$\beta_{2,g}$ captures deviation from CN-proportional expression,

$\beta_{3,g}$ represents expression contributed by non-cancer (normal) cells,

$c_{g,n}$ is a gene-dosage component relative to diploid state,

$d_{g,n}$ is a signed gene-dosage deviation term, with positive values indicating gains and negative values indicating losses,
ϵ is an estimation error bias.
An identity link is used to preserve an interpretable decomposition of counts into additive components.
This parameterization allows to separate genes whose expression is proportional to CN from those exhibiting dosage compensation or amplification-specific regulatory effects. Conceptually, the $c_{g,n}$ term reflects how many gene copies are present, whereas $d_{g,n}$ captures how transcriptional output deviates from CN expectations.
Negative values of $\beta_{2,g}$ indicate dosage compensation, whereas positive values identify genes with hyperactivation (genes with expression level higher than CN-driven expectations).
To ensure stable inference under identity-link negative binomial model, we used weakly informative priors to regularize estimation without constraining biologically plausible effects. Gene-specific dosage coefficients $\beta_{1,g}$ and non-cancer expression coefficients $\beta_{3,g}$ were constrained to be positive with log-normal priors:

$$\beta_{1,3,g} \sim \mathrm{LogNormal}(0, 1) \qquad (4)$$

For the deviation coefficient $\beta_{2,g}$ was assigned zero-centered normal prior to allow both positive (hyperactivation) and negative effects (compensation):

$$\beta_{2,g} \sim \mathrm{Normal}(0, 0.2) \qquad (5)$$

The overdispersion parameter θ_g was constrained to be positive and was assigned exponential prior.
Posterior inference of model parameters was performed independently for each gene using Hamiltonian Monte Carlo [3] as implemented in Stan probabilistic framework (via CmdStanPy) [4] .

Summary statistics and interpretation

For each gene we used posterior estimates of $\beta_{1,g}$ and $\beta_{2,g}$ to calculate summary statistics.
To obtain a scale-invariant measure of deviation, the deviation coefficient $\beta_{2,g}$ was normalized by dosage-sensitivity coefficient $\beta_{1,g}$, defining a compensation ratio:

$$\beta^*_{2,g} = \frac{\mathbb{E}[\beta_{2,g}]}{\mathbb{E}[\beta_{1,g}]} \qquad (6)$$

Statistical significance (p-value) of deviation from dosage proportionality was calculated using a z-score derived from the posterior distribution of $\beta_{2,g}$:

$$z_g = \frac{\mathbb{E}[\beta_{2,g}]}{\mathrm{sd}(\beta_{2,g})} \qquad (7)$$

from which two-sided p-values were computed under a standard normal approximation.
To account for statistical uncertainty in downstream analyses, the normalized deviation $\beta^*_{2,g}$ was shrunk toward zero using the corresponding p-value:

$$C_g \approx \beta^*_{2,g} \cdot (1 - pvalue_g) \qquad (8)$$

This shrinkage was applied such that genes with weak evidence for deviation were down-weighted, while genes with strong posterior support retained large-magnitude scores. As a result, the final compensation score integrates both effect size and statistical confidence into a single interpretable metric.

Gene-dosage class assignment

Genes were classified as dosage-sensitive (DSG), dosage-compensated (DCG), or hyperactivated (HYPER) based on the sign and magnitude of the normalized deviation score C_g.

- DCGs: genes showing high negative deviation from CN proportionality $C_g \leq -\tau$, where τ is a minimum effect-size threshold.
- Hyperactivated genes: genes showing high positive deviation from CN proportionality: $C_g \geq \tau$.
- DSGs: genes with small deviation magnitude: $|C_g| \leq \tau$.

Model agreement between DeConveil and NB regression

To assess consistency between gene classifications derived from `DeConveil` and the NB regression framework, we performed a model agreement analysis on genes identified by both methods. `DeConveil`-derived gene classes (DSGs, DIGs and DCGs), were defined using absolute log2 fold-change (|LFC|) and adjusted p-value thresholds. NB regression classifications (DSG, DCG, HYPER, or OTHER) were derived as described above.
To enable comparison across methods, effect-size thresholds were aligned by applying matched cutoffs to the `DeConveil` |LFC| and NB regression compensation score C_g.
Two effect-size regimes were considered: a moderate effect regime, defined by |LFC| > 0.3 in `DeConveil` and $|C_g| > 0.3$ in NB regression, and a strong effect regime, defined by |LFC| > 1.0 in `DeConveil` and $|C_g| > 1.0$ in NB regression. Gene classifications were recomputed under each regime prior to concordance analysis.
For genes common to both analyses, concordance was summarized by constructing contingency tables of `DeConveil` versus NB regression class assignments. Within each `DeConveil` class, row-normalized proportions were calculated to quantify the fraction of genes assigned to each NB class.

Entropy-based analysis of dosage-compensated genes

To characterize heterogeneity within `DeConveil` defined DCGs, we analyzed posterior class probability distributions obtained from the NB regression model. For each DCG, posterior probabilities corresponding to NB classes were normalized to sum to one. For each gene were computed two summary metrics: (i) dominance, defined as the maximum posterior probability across NB classes, and (ii) normalized Shannon entropy [5], defined as

$$H_g = -\frac{1}{\log K}\sum_{k=1}^{K} p_{g,k}\log p_{gk} \qquad (9)$$

where $p_{g,k}$ is the posterior probability of gene *g* belonging to NB class *k,* and *K* is the number of NB classes. Entropy values range from 0 to 1, indicating dominance of a single class or maximal uncertainty across classes respectively.

Based on dominance and entropy metrics, `DeConveil` DCGs were further subdivided into interpretable subtypes:

- Pure DCGs were defined as genes classified as DCGs by both NB regression and `DeConveil` or showing high entropy (≥ 0.7), indicating heterogeneous dosage behavior across classes.
- Directional DCGs were defined as `DeConveil`-classified DCGs whose NB regression posterior distribution was dominated by a single directional class (e.g. DSG), with a maximum posterior probability ≥ 0.6. Although these genes are classified as dosage-compensated by `DeConveil` in tumor-normal comparisons, the NB tumor-only model shows that these genes display a predominantly directional relationship between CN and expression. This behavior suggests that dosage-compensation is not uniform across modeling frameworks or effect-size regimes.
- Ambiguous DCGs were defined as genes with intermediate entropy values (0.3–0.7) and no dominant NB class.

Selection of representative genes for visualization

Representative genes for visualization were selected separately for each gene-dosage class. Within each class, genes were ranked by the absolute value of the corresponding effect-size measure (log2 fold change for `DeConveil` classes or compensation score for NB regression classes), and the gene whose effect size was closest to the class median was chosen. Selected genes were used solely for illustrative purposes.

References

1. Agresti A. Foundations of Linear and Generalized Linear Models. John Wiley & Sons; 2015.

2. Hilbe JM. Negative Binomial Regression. Cambridge University Press; 2011.

3. Thomas S, Tu W. Learning Hamiltonian Monte Carlo in R. Am Stat. 2021;75: 403–413.

4. Carpenter B, Gelman A, Hoffman MD, Lee D, Goodrich B, Betancourt M, et al. Stan: A Probabilistic Programming Language. J Stat Softw. 2017;76. doi:10.18637/jss.v076.i01

5. Gao J, Hu J, Tung W-W. Entropy measures for biological signal analyses. Nonlinear Dynamics. 2011;68: 431–444.
